# Next-generation transgenic mice for optogenetic analysis of neural circuits

**DOI:** 10.3389/fncir.2013.00160

**Published:** 2013-11-26

**Authors:** Brent Asrican, George J. Augustine, Ken Berglund, Susu Chen, Nick Chow, Karl Deisseroth, Guoping Feng, Bernd Gloss, Riichiro Hira, Carolin Hoffmann, Haruo Kasai, Malvika Katarya, Jinsook Kim, John Kudolo, Li Ming Lee, Shun Qiang Lo, James Mancuso, Masanori Matsuzaki, Ryuichi Nakajima, Li Qiu, Gregory Tan, Yanxia Tang, Jonathan T. Ting, Sachiko Tsuda, Lei Wen, Xuying Zhang, Shengli Zhao

**Affiliations:** ^1^Department of Neurobiology, Duke University Medical CenterDurham, NC, USA; ^2^Center for Functional Connectomics, Korea Institute of Science and TechnologySeoul, South Korea; ^3^Laboratory of Synaptic Circuitry, Program in Neuroscience and Behavioral Disorders, Duke-NUS Graduate Medical SchoolSingapore, Singapore; ^4^A^*^STAR/Duke-NUS Neuroscience Research PartnershipSingapore, Singapore; ^5^Department of Physiology, Yong Loo Lin School of Medicine, National University of SingaporeSingapore, Singapore; ^6^Marine Biological LaboratoryWoods Hole, MA USA; ^7^Lee Kong Chian School of Medicine, Nanyang Technological UniversitySingapore, Singapore; ^8^Institute of Molecular and Cell BiologySingapore, Singapore; ^9^NUS Graduate School for Integrative Sciences and EngineeringSingapore, Singapore; ^10^Department of Bioengineering, Stanford UniversityPalo Alto, CA, USA; ^11^Department of Brain and Cognitive Sciences, McGovern Institute for Brain Research, Massachusetts Institute of TechnologyCambridge, MA, USA; ^12^Division of Brain Circuits, National Institute for Basic Biology and The Graduate University of Advanced Studies (Sokendai)Myodaiji, Okazaki, Japan; ^13^CREST, Japan Science and Technology AgencySaitama, Japan; ^14^Laboratory of Structural Physiology, Faculty of Medicine, Center for Disease Biology and Integrative Medicine, University of TokyoTokyo, Japan

**Keywords:** optogenetics, channelrhodopsin, photostimulation, photoinhibition, cerebellum, cortex, hippocampus, pons

## Abstract

Here we characterize several new lines of transgenic mice useful for optogenetic analysis of brain circuit function. These mice express optogenetic probes, such as enhanced halorhodopsin or several different versions of channelrhodopsins, behind various neuron-specific promoters. These mice permit photoinhibition or photostimulation both *in vitro* and *in vivo*. Our results also reveal the important influence of fluorescent tags on optogenetic probe expression and function in transgenic mice.

## Introduction

One of the fundamental goals of neuroscience is to understand how the high-order functions of the brain emerge from the complex networks formed by many types of neurons with diverse genetic, physiological, and anatomical properties. Optogenetic tools provide unprecedented opportunities for approaching this goal by causally linking the activity of specific types of neurons or neural circuits to behavioral output.

Several optogenetic actuators have been identified that allow photostimulation or photoinhibition of genetically defined populations of neurons with high temporal and spatial resolution. Among these, the light-gated ion channel channelrhodopsin-2 from *Chlamydomonas reinhardtii* (ChR2) (Nagel et al., 2003; Boyden et al., 2005) and channelrhodospin-1 from *Volvox carteri* (VChR1) (Zhang et al., 2008), as well as chimeric constructs such as C1V1 (Yizhar et al., 2011), have been developed for photostimulation. Among many other applications, the ability to selectively photostimulate defined populations of neurons enables high-speed mapping of the spatial organization of circuits by photostimulating presynaptic neurons with a scanned laser beam while using electrophysiology to detect postsynaptic responses in downstream neurons (Petreanu et al., 2007; Wang et al., 2007; Mao et al., 2011; Kim et al., in revision).

Similarly, probes have been developed to enable optogenetic photoinhibition of neurons. The first example of this class of probes was the light-driven chloride pump, halorhodopsin, from *Natronomonas pharaonis* (NpHR; Han and Boyden, 2007; Zhang et al., 2007) and its improved versions eNpHR 2.0 and eNpHR 3.0 (Gradinaru et al., 2008, 2010; Zhao et al., 2008), as well as light-driven proton pumps such as archaerhodopsin-3 from *Halorubrum sodomense* (Arch; Chow et al., 2010) and bacteriorhodopsin (Gradinaru et al., 2010) have been harnessed for photoinhibition.

In order to be useful for neural circuit breaking, these optogenetic probes must be highly expressed in cell-type specific manner. Although *in utero* electroporation (Petreanu et al., 2007; Huber et al., 2008) and virus-based introduction of optogenetic probes (for examples, see Boyden et al., 2005; Ishizuka et al., 2006; Atasoy et al., 2008; Kuhlman and Huang, 2008; Tsai et al., 2009) enable high-copy expression in mammalian systems, these strategies are limited by incomplete coverage of target neuronal populations, variable expression levels across cells, and difficulty in identifying a cell-type specific promoter with an appropriate size for viral packaging. These limitations can be overcome by generating transgenic animals with targeted expression of optogenetic probes. Transgenic animal lines offer the important advantage of reproducible and stable patterns of optogenetic probe expression in defined neuronal populations within all individuals of the line across generations.

ChR2 and NpHR have been inserted downstream of a variety of different promoters including *Thy1* (Arenkiel et al., 2007; Wang et al., 2007; Zhao et al., 2008), *Omp* (Dhawale et al., 2010) and *Orexin* (Tsunematsu et al., 2011). Because this strategy is based on random insertion of a transgene, which can cause problems due to multiple insertion sites, it is becoming more popular to use bacterial artificial chromosomes (BAC) containing the gene for optogenetic probes along with cell-type specific promoters and necessary regulatory elements for transgene expression. ChR2 has been successfully expressed in such BAC-based transgenic mice, under regulation by the *Vglut2* (Hägglund et al., 2010), *Chat* (Ren et al., 2011; Zhao et al., 2011), *VGAT*, *TPH2*, and *Pvalb* (Zhao et al., 2011) promoters.

A more flexible approach to generating optogenetic mice comes from crossing existing Cre driver lines with lines containing transgenes for optogenetic probes downstream of a floxed stop cassette. This approach takes advantage of the hundreds of cell-type specific Cre driver lines that are available. For conditional expression of optogenetic probes from a defined genomic locus, the Cre/loxP system has been proven an efficient approach to achieve genetic targeting of optogenetic probes with high levels of expression. To generate a Cre-responsive allele, the gene for the optogenetic probe is inserted into a modified *Rosa26* locus under the control of a floxed stop cassette, with expression driven by a strong and ubiquitous promoter (Madisen et al., 2010). Recently such lines were developed to allow conditional expression of ChR2, Arch, or eNpHR: after breeding those mice with *Cre* driver lines, the optogenetic probes are specifically and robustly expressed in a variety of neuron types (Madisen et al., 2012). By using a tamoxifen-sensitive Cre mouse line, it has even been possible to precisely control the timing of ChR2 expression (Katzel et al., 2011). The tetracycline transactivator (tTA)-tetracycline operator (tetO) promoter system is an alternative bigenic approach to generating transgenic optogenetic mice (Chuhma et al., 2011; Tanaka et al., 2012).

Expansion of optogenetic mapping of neural circuits requires the creation of new tools that expand the number of neuronal targets available for photostimulation/photoinhibition, as well as permit combination of tools in the same animal. With these goals in mind, we have used a variety of strategies to generate additional mouse lines. These new transgenic lines take advantage of known promoter sequences, a previously described BAC transgenic strategy, or a combination of existing transgenic lines for conditional expression. These mice provide new opportunities for optogenetic manipulation of neuronal activity and also provide some useful technical guidance for engineering future optogenetic mice. This paper describes these new mice and characterizes their utility for optogenetic analysis of neural circuitry, with emphasis on their use for high-speed photostimulation-mediated circuit mapping (Petreanu et al., 2007; Wang et al., 2007; Mao et al., 2011; Kim et al., in revision).

## Materials and methods

### Transgenic mice

Transgenic mice expressing optogenetic actuators in specific, genetically-defined populations of neurons were prepared using either conventional targeting vectors, as described in Wang et al. (2007), or using a BAC transgenic strategy, as described in Zhao et al. (2011). The specific features of the various lines described in this paper are shown in Table 1. Note that many of these have been given to Jackson Labs (JAX) for commercial distribution. Transgenic mice were backcrossed to C57BL/6 and hemizygous transgenic mice were used in our experiments. PCR-based genotyping of mice was done as described in Wang et al. (2007); see Table 1 for the primers used for genotyping each mouse line. PCP2-ChR2-H134R mice were generated by crossing hemizygous PCP2-cre transgenic mice [(Pcp2-cre)2Mpin/J; Jackson Labs] (Barski et al., 2000) to hemizygous mice expressing floxed ChR2-H134R [B6;129S-Gt(ROSA)26Sor^*tm32*(*CAG*−*COP*4∗*H*134*R*/*EYFP*)*Hze*/*J*^] (Madisen et al., 2012) and selecting mice positive for both transgenes. Mice were maintained with free access to food and water under a 12 h light/dark cycle. All experimental procedures were approved by and conducted in accordance with the ethical guidelines of the animal care and use committees of our respective institutions.

**Table 1:** **Optogenetic mouse lines used for this paper**.

**Mouse line**	**Promoter actuator**	**Optogenetic**	**Fluorescent tag**	**Cellular targets**	**JAX stock number**	**PCR primers (forward, reverse)**
Thy1-NpHR	Thymus cell antigen 1 (Thy1.2)	Halobacteria halorhodopsin Zhao et al., 2008	EYFP	Projection neurons	–	TCT GAG TGG CAA AGG ACC TTA GG
						TCC ACC AGC AGG ATA TAC AAG ACC
Thy1-NpHR 2.0	Thy 1.2	Enhanced halorhodopsin Gradinaru et al., 2008; Zhao et al., 2008	EYFP	Projection neurons	012332 (line 2)	TCT GAG TGG CAA AGG ACC TTA GG
					012334 (line 4)	TCC ACC AGC AGG ATA TAC AAG ACC
Thy1-VChR1	Thy 1.2	*Volvox* channelrhodopsin-1 Zhang et al., 2008	EYFP	Projection neurons	012344 (line 4)	TCT GAG TGG CAA AGG ACC TTA GG
					012348 (line 8)	TGT GAG GTT GCT CAG ATG G
Thy1-ChR2-YFP	Thy 1.2	*Chlamydomonas* channelrhodopsin-2 Boyden et al., 2005	EYFP	Projection neurons	007612	TCT GAG TGG CAA AGG ACC TTA GG
						GAA GAT GAC CTT GAC GTA TCC G
PV-ChR2-mCherry	Parvalbumin	*Chlamydomonas* channelrhodopsin-2	mCherry	PV-positive (usually GABAergic) neurons	–	–
PV-hChR2-YFP	Parvalbumin	Mammalian codon optimized channelrhodopsin-2	EYFP	PV-positive (usually GABAergic) neurons	–	CTT TTC GCA CTT GCT CTG C
						GCA AGG TAG AGC ATA GAG GG
Thy1-hChR2-tdTomato	Thy 1.2	Mammalian codon optimized channelrhodopsin-2	tdTomato	Projection neurons	–	TCT GAG TGG CAA AGG ACC TTA GG
						GCA AGG TAG AGC ATA GAG GG
PV-hChR2(H134R)-EYFP (line 15)	Parvalbumin	Mammalian codon optimized channelrhodopsin-2 with gain of function H134R mutation	EYFP	PV-positive (usually GABAergic) neurons	012355	CTT TTC GCA CTT GCT CTG C
						GCA AGG TAG AGC ATA GAG GG
PCP2-Cre-ChR2	Purkinje cell protein 2	Mammalian codon optimized channelrhodopsin-2 with H134R mutation	EYFP	Cerebellar Purkinje cells	004146 (Pcp2 Cre) X	GCG GTC TGG CAG TAA AAA CTA TC
					012569 (floxed ChR2)	GTG AAA CAG CAT TGC TGT CAC TT
PV-Cre-ChR2	Parvalbumin	Mammalian codon optimized channelrhodopsin-2 with H134R mutation	EYFP	PV-positive (usually GABAergic) neurons	008069 (PV-Cre) X	GCG GTC TGG CAG TAA AAA CTA TC
					012569 (floxed ChR2)	GTG AAA CAG CAT TGC TGT CAC TT

### Histological characterization of transgene expression

Histology was used to characterize brain expression of optogenetic probes. For this purpose, adult transgenic mice were euthanized with an overdose of halothane or isoflurane and transcardially perfused with 0.1 M phosphate buffer saline (pH 7.4) followed by 4% paraformaldehyde. The brain was removed and stored at 4°C in the fixative overnight. The brain was then sectioned into 50-μm-thick slices on a freezing microtome. Low magnification fluorescence images were obtained on an upright epi-fluorescence microscope (Nikon Eclipse E600-FN or Zeiss AxioImager). Higher magnification images were obtained by laser-scanning confocal microscopes (Leica TCS SP2, Nikon A1Rsi or Zeiss LSM510 META). In some cases, live slices from PV-ChR2-EYFP or Thy1-hChR2-tdTomato mouse brains were prepared as described below and then imaged on a 2-photon microscope (Olympus FV-1000).

### Whole-cell patch clamp recording from brain slices

Brain slices were prepared from transgenic mice aged 2 weeks to 3 months, using conventional methods (Pettit and Augustine, 2000; Wang et al., 2007). In brief, isolated brains were sliced (200–350 μm thick parasagittal or coronal sections) in a cold artificial cerebrospinal fluid (ACSF) containing (in mM): 125 NaCl, 2.5 KCl, 1.25 NaH_2_PO_4_, 26 NaHCO_3_, 20 d(+)-glucose, 2–2.5 CaCl_2_ and 1–1.3 MgCl_2_ (some experiments included 0.4 mM ascorbic acid) or a high-sucrose ACSF containing: 240 Sucrose, 26 NaHCO_3_, 2.5 KCl, 1.0 CaCl_2_, 4 MgCl_2_, 1.25 NaH2PO4, and 10 d(+)-glucose (some experiments included 3 myo-inositol and 1 kynurenic acid), pH 7.4, by gassing with 95% O_2_/5% CO_2_. Slices were transferred to an incubation chamber containing oxygenated ACSF and incubated at 36°C for 30 min and at least 1 h at room temperature prior to use.

Whole-cell patch clamp recordings were performed at room temperature (21–24°C) or 32°C (for the case of Figures 8A–C) under an upright microscope (Nikon Eclipse E600-FN or Olympus BX61WI) in a recording chamber perfused with 95% O_2_/5% CO_2_ aerated extracellular solution (ACSF). Bicuculline (10 μM; Sigma, St. Louis, MO) or GABAzine (SR-95531; 10 μM; Sigma, St. Louis, MO) or 50 μM picrotoxin (Wako, Osaka, Japan), CNQX (10 μM; Sigma) and APV (2-amino-5-phosphonovaleric acid; 50 μM; Sigma) were sometimes added to the ACSF to block synaptic transmission.

Neurons expressing the various optogenetic probes were identified by their fluorescence; for this purpose, the following filter sets were employed: EYFP—465–495 nm excitation, 505 nm dichroic, 515–555 nm emission; mCherry—528–553 nm excitation, 565 nm dichroic, 590–650 nm emission. Fluorescence was detected with a CoolSNAP-fx camera (Photometrics, Tucson, AZ) or with an Olympus FV1000-MPE laser-scanning microscope. Whole-cell patch-clamp recordings were made from these neurons using pipettes (2–7 MΩ) filled with internal solution containing (in mM): 130 K-gluconate, 2 NaCl, 4 MgCl_2_, 20 HEPES, 4 Na_2_ATP, 0.4 Na_3_GTP, 0.25–0.5 EGTA, and 0–0.5 Na_2_ phosphocreatine, pH 7.25 with 1 M KOH; 290–295 mOsm. For experiments involving 2-photon imaging of neurons in slices, internal solutions also contained the fluorescent dye Alexa Fluor 594 (Invitrogen). In these experiments, an Olympus FV-1000 microscope was used for 2-photon imaging (790 nm excitation) of neuronal structure. Unless otherwise indicated, all current measurements were made at a holding potential of −70 mV. When appropriate, a junction potential of 10 mV was taken into account when reporting membrane potentials. Electrical responses were acquired with a patch-clamp amplifier (Multiclamp 700B, Axoclamp 1B, or Axoclamp 2B; all Molecular Devices), digitized at 20 kHz via a Digidata 1440A interface (Molecular Devices), acquired using pClamp software (Molecular Devices), and analyzed using Clampfit, Igor Pro, and/or Origin 8 software.

### Photostimulation/photoinhibition

Two different styles of light stimuli were employed to photostimulate or photoinhibit neurons in our experiments. For wide-field illumination, an upright epi-fluorescence microscope (Nikon Eclipse E600-FN) was used to illuminate the entire width of the microscope field. In such experiments, an arc lamp was used to provide light which was then filtered by various bandpass filters to activate ChR2 (465–495 nm), NpHR and eNpHR2.0 (545–585 nm), and VChR1 (528–553 nm). Light pulse duration was controlled by an electronic shutter (Uniblitz T132; Vincent, Rochester, NY) and intensity was adjusted by neutral density filters. In a few experiments, a light-emitting diode (Prizmatix UHP-LED-460) provided monochromatic light (460 nm) to activate ChR2.

In other experiments, optogenetic probes were activated by small spots of laser light as previously described in Wang et al. (2007). These experiments used a FV-1000MPE laser scanning microscope (Olympus) equipped with a 25× (1.05 NA) water-immersion objective. In brief, an area of ~500 × 500 μm was scanned with a 405 nm laser spot (typically 4 ms duration) in a 32 × 32 array of pixels. The laser spot was scanned in a pseudorandom sequence, to avoid activation of adjacent pixels, while cellular responses were simultaneously measured with whole-cell patch clamp recordings.

For *in vivo* photoinhibition (Figure 5), transgenic mice expressing eNpHR2.0 (aged 3–6.5 months) were used. To fasten the mice under the microscope, headplates were attached to the skull, as described in Hira et al. (2013), and the mice were allowed to recover from this surgery for several days. Mice were then anesthetized with isoflurane (0.7–1.1%), the skull was opened, and the mice were placed under the microscope (Olympus FV1000-MPE laser-scanning microscope). Electrical recordings were made with an Elgiloy microelectrode with an impedance of 2 MΩ (TM33B20KT; WPI) that was inserted into the left caudal forelimb area (CFA; Hira et al., 2013) at a depth of ~600 μm beneath the cortical surface. Electrode signals were amplified (DAM-80 amplifier; WPI), filtered at 1–300 Hz or 500–1000 Hz (SIM900; Stanford Research Systems, Sunnyvale, CA), and sampled at 5 kHz (FV1000 system; Olympus).

Spot photostimulation was performed in anesthetized mice using a green laser (559 nm; 3.2 mW) and an upright microscope (Olympus BX61WI). The area of photostimulation was 2.6 × 2.6 mm when using a 5× MPL objective (0.10 NA, Olympus) and included the cortical surface of the left CFA. This area was divided into two-dimensional 32 × 32 pixel arrays and each pixel was then individually illuminated with the laser scanning system, as described above. To reconstruct a local field potential (LFP) map, the LFP signals were filtered at 1–300 Hz. For each pixel, the mean LFP signal measured for 50 ms before the start of the illumination was subtracted from the maximal signal during the illumination (20 ms) at each pixel.

To examine photoinhibition of limb movements, forelimb movement was evoked by intracortical microstimulation and detected by a CCD camera. Stimulation was performed with a tungsten microelectrode (2 MΩ impedance; TM33B20KT; WPI) inserted to the right CFA at a depth of ~600 μm beneath the cortical surface. Thirty minutes after isoflurane anesthesia, the right CFA was stimulated with a 150 ms train of 0.5 ms cathodal pulses of 60 μA at 333 Hz. While the electrical stimulation was repeated at 1 Hz, the whole field of view (3.7 mm diameter), including the cortical surface of the right CFA, was repeatedly and uniformly illuminated for ~10 s with an orange laser (594 nm; MGL-N-593.5, Changchun New Industries Optoelectronics Tech.) that was introduced to the microscope through a large-core fiber. Laser intensity on the cortical surface was ~100 mW (9 mW/mm^2^).

## Results

### Improved photoinhibition with enhanced halorhodopsin

We begin characterization of our new optogenetic mouse lines by describing NpHR transgenic mice. Neurons from transgenic mice expressing the first-generation halorhodopsin (NpHR) exhibited light-induced photocurrents and photoinhibition of action potential firing (Zhao et al., 2008). However, in these mice some halorhodopsin was retained within the endoplasmic reticulum, yielding neurons with swollen dendrites and axons (Zhao et al., 2008). To avoid problems with intracellular trafficking of NpHR, we took advantage of an enhanced halorhodopsin (eNpHR2.0) that has an added ER export motif and an improved signal peptide sequence to enhance membrane trafficking (Gradinaru et al., 2008; Zhao et al., 2008).

**Figure 1:**
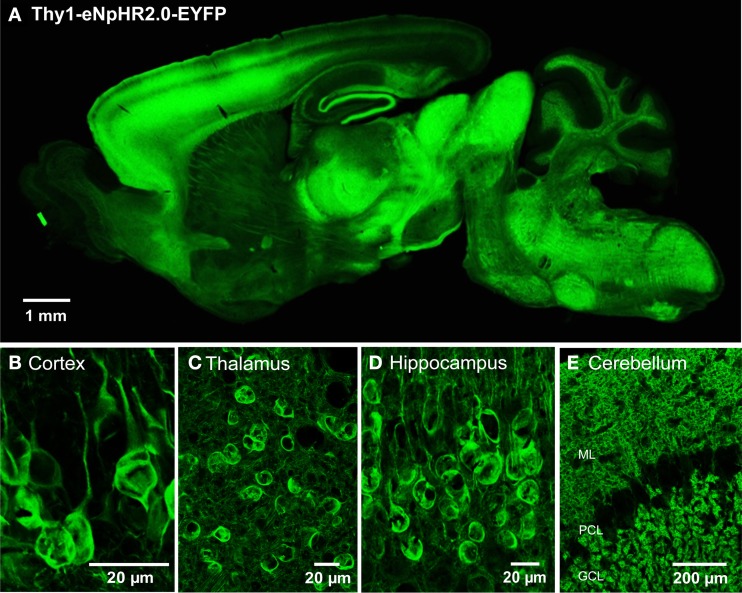
**Expression of eNpHR2.0 in Thy1-eNpHR2.0 line 4 transgenic mouse brain. (A)** Sagittal section from an adult transgenic mouse brain (Thy1-eNpHR2.0 line 4). **(B)** Expression of eNpHR2.0 in cortical pyramidal cells (line 2). **(C)** Expression of eNpHR2.0 in anteroventral thalamic nucleus (line 2). **(D)** Expression of eNpHR2.0 in CA1 pyramidal cells. **(E)** Expression of eNpHR2.0 in cerebellar mossy fibers (line 2); ML, molecular layer; PC, Purkinje cell layer; GCL, granule cell layer.

We engineered two transgenic mouse lines, lines 2 and 4, that used the Thy1.2 promoter to yield neuron-specific expression of eNpHR2.0. The enhanced yellow fluorescent protein, EYFP, was fused to eNpHR2.0 to allow us to visualize its brain distribution and subcellular localization. These two lines had high levels of eNpHR2.0 expression in multiple regions of the brain (Figure 1A). eNpHR2.0-positive cells included neurons in various regions of the amygdala, midbrain, and lower brainstem, pyramidal cells in layer 5 of the cortex (Figure 1B), cells in the anteroventral thalamic nucleus (Figure 1C), granule cells in the dentate gyrus and pyramidal cells in hippocampal CA1 region (Figure 1D), and mossy fibers in the granule cell layer of the cerebellum (Figure 1E). A detailed description of the cellular expression of eNpHR in line 2 is provided in Table 2 below. In all cases, eNpHR2.0 appeared to be efficiently targeted to the plasma membrane, as indicated by staining around the circumference of cell bodies, and the absence of cell swelling or punctate fluorescent structures suggested a lack of retention within the endoplasmic reticulum.

**Table 2:**
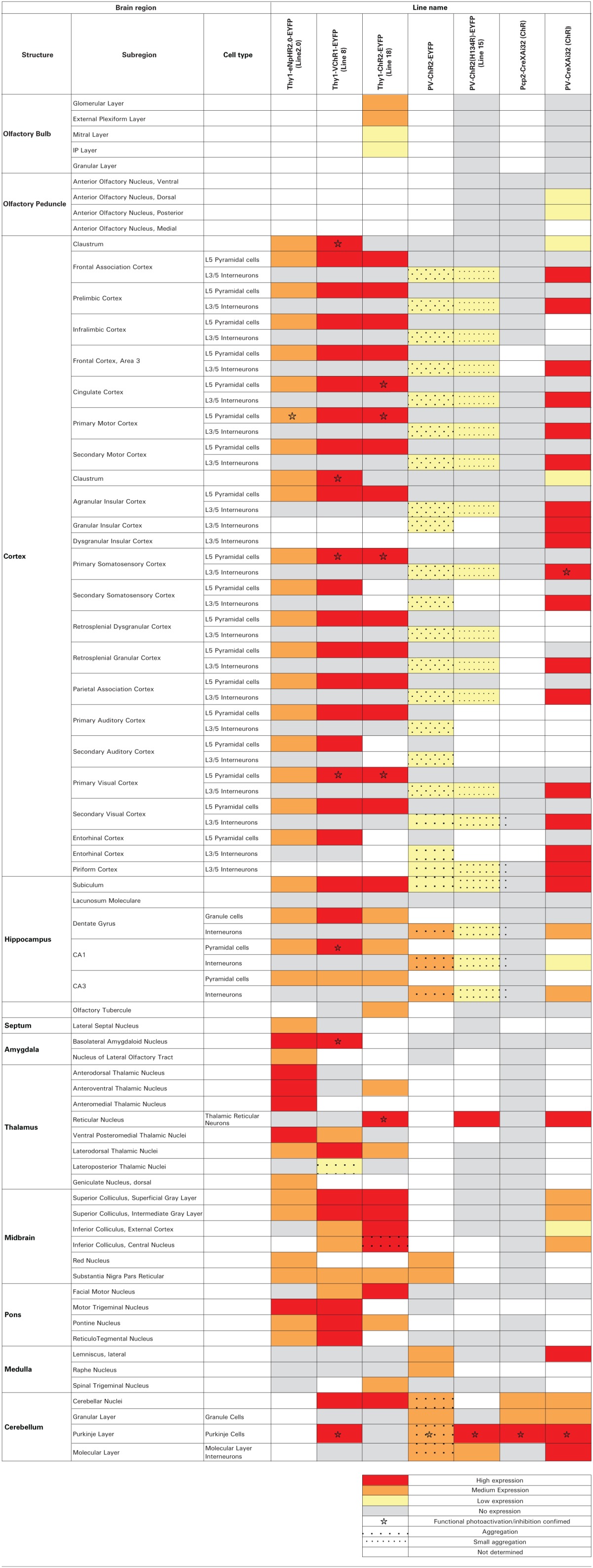
**Patterns of expression of optogenetic probes in transgenic mouse lines**.

To determine whether eNpHR2.0 expression altered the functional characteristics of neurons, we measured the electrophysiological properties of pyramidal neurons in cortical layer 5 from Thy1-eNpHR2.0 line 2 (*n* = 10) mice and wild-type controls (*n* = 5). Resting membrane potentials were not significantly different between non-expressing (−70.0 ± 1.3 mV) and eNpHR2.0-expressing neurons (−76.4 ± 1.7). Likewise, action potential properties were similar between the two types of neurons (data not shown). Taken together, these data indicate that eNpHR2.0 does not affect neuronal electrophysiological properties in the absence of light.

**Figure 2:**
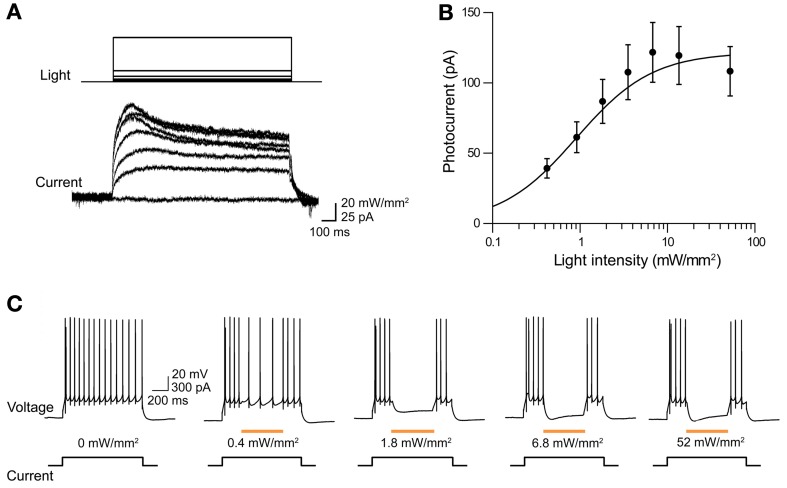
**Illumination evokes outward photocurrents and inhibition of action potential firing in cortical layer 5 pyramidal cells from Thy1-eNpHR2.0 line 4 mice. (A)** 1-s long light flash (575 nm) induced photocurrents that increased in amplitude with brighter light flashes. **(B)** Relationship between photocurrent amplitude and light intensity. Points indicate means ± sem of eighteen neurons and curve is a fit of the Hill equation. **(C)** Light pulses of varying intensity elicited graded hyperpolarization and inhibition of action potentials firing in a pyramidal cell.

The ability of eNpHR2.0 to photoinhibit neuronal activity was examined in cortical layer 5 pyramidal cells (Figure 2). In these experiments, light spots illuminating large areas of the slices (≈0.4 mm^2^) were used. At a holding potential of −70 mV, 1-s long light pulses (545–585 nm) evoked outward photocurrents that peaked within 100 ms (Figure 2A). Responses were recorded from a total of 17 eNpHR2.0-expressing layer 5 cortical pyramidal cells from postnatal day 17 (P17) to P21 mice. The maximum photocurrent induced by light was 121 ± 21.3 pA (mean ± sem here and subsequently). In contrast, no photocurrents were generated by illumination of neurons in slices from wild-type mice.

The magnitude of the photocurrents mediated by eNpHR2.0 depended upon light intensity, with stronger illumination yielding larger currents (Figure 2A). This is caused by progressive activation of more eNpHR2.0 pumps as the light intensity increases. The relationship between light intensity and peak amplitude of the photocurrent (Figure 2B) could be described by the Hill equation:

$$Y=I_{\text{max}}\frac{X^{n}}{K^{n}+X^{n}}$$

where *X* is light luminance, *Y* is photocurrent amplitude, and *K* represents the light level where the photocurrent was half-maximal (0.79 ± 0.12 mW/mm^2^). *I*_max_, the maximum current amplitude, was 119 ± 5.7 pA and *n*, the Hill coefficient, was 1.39 ± 0.12. The Hill coefficient of close to 1 indicated that a single photon, rather than multiple photons, was sufficient for activation of a single eNpHR2.0 molecule (Kolbe et al., 2000). Similar Hill coefficients were observed for all of the other optogenetic probes described in this paper, consistent with their known requirements for absorption of a single photon for activation.

**Figure 3:**
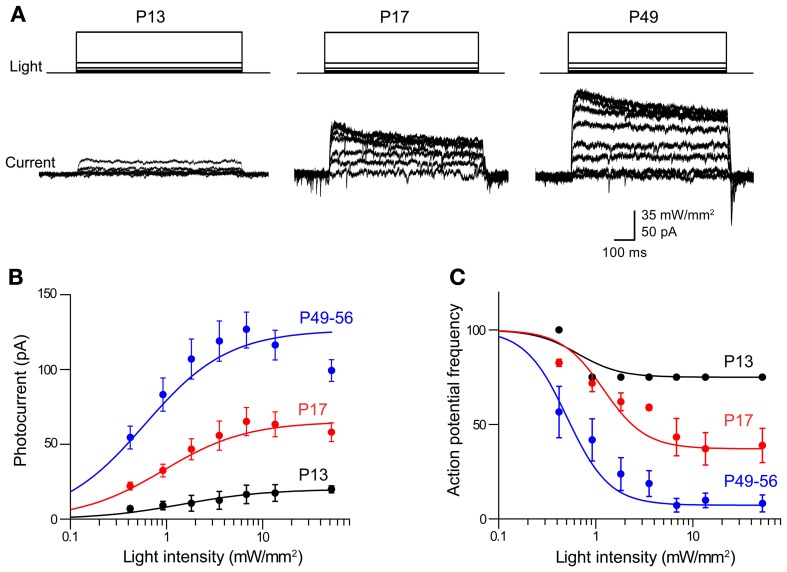
**Photoinhibition increases over development in Thy1-eNpHR2.0 mouse lines. (A)** Photocurrents induced by a series of light flashes (575 nm, 1 s duration) in pyramidal neurons in cortical slices from Thy1-eNpHR2.0 mice of the indicated ages. **(B)** Relationship between photocurrent amplitude and light intensity determined for mice of different ages. Points indicate means ± sem (P13: *n* = 4; P17: *n* = 9; P49–56: *n* = 7). Curves are fits of the Hill equation. **(C)** Relationship between light intensity and degree of inhibition of action potential firing at the indicated ages. Photoinhibition is greater in older mice, due to a higher level of eNpHR2.0 expression.

To examine the effects of eNpHR2.0 activation on neuronal excitability, pyramidal neurons were depolarized with current pulses (200 pA, 1 s duration) to evoke trains of action potentials. Illumination (500 ms duration) in the midst of these current pulses reduced AP frequency, with brighter light flashes causing a higher degree of photoinhibition (Figure 2C). Fits of the Hill equation (see Figure 3C below) indicated a half-maximal light intensity of 0.42 ± 0.02 mW/mm^2^. The maximum reduction of action potential frequency by light flashes was 85.3 ± 0.9% under these conditions, though of course this value depends upon the amount of depolarizing current applied. Together these data demonstrate that eNpHR2.0 is an effective tool for silencing neuronal activity when genetically targeted and chronically expressed in transgenic mice.

In adults (P56) from line 4, peak photocurrents in cortical pyramidal neurons were 126 ± 12.0 pA, which is not significantly different (*p* > 0.05, two-tailed *t*-test) from the values of 165 ± 37.1 pA determined for line 2 pyramidal neurons at this age. However, the magnitude of the eNpHR2.0-mediated photocurrents sharply varied according to the age of the mouse. Photocurrent magnitude increased with age, ranging from very small at P13 (Figure 3A, left) to substantially larger at P49–56 (Figure 3A, right). Figure 3B characterizes the time course of eNpHR2.0 expression by comparing the relationship between light intensity and photocurrent magnitude at different ages. While the shape of this relationship was relatively constant over the course of development, the maximum photocurrent increased several-fold between P13 and P56. This indicates that eNpHR2.0 expression increases steeply over the first few weeks after birth. Using the same paradigm depicted in Figure 2C, we could determine how this progressive expression of eNpHR2.0 affects photoinhibition of action potential firing. In P13 neurons, illumination of pyramidal cells expressing eNpHR2.0 minimally reduced action potential firing, even at maximal light intensities (Figure 3C). In contrast, photoinhibition was more effective in P17 neurons and by P49–56, eNpHR2.0 activation could potently inhibit neuronal firing. This age-dependent increase in the degree of photoinhibition presumably is due to the higher level of eNpHR2.0 expression depicted in Figure 3B.

**Figure 4:**
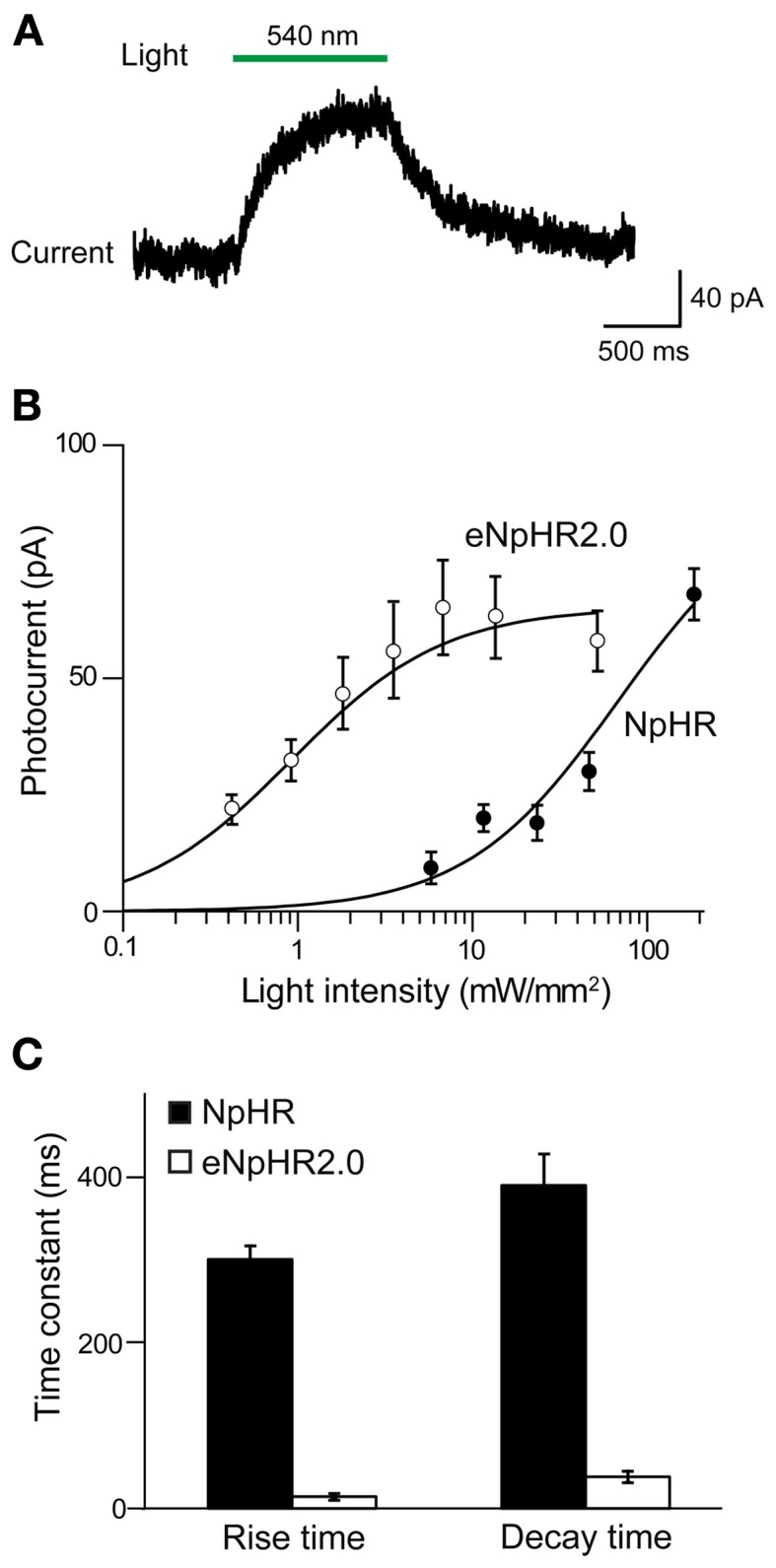
**Comparison of photocurrents generated by NpHR and eNpHR2.0. (A)** Illumination (1 s duration; 186 mW/mm^2^) evokes outward photocurrent in pyramidal cell from Thy1-NpHR mice. **(B)** Comparison of photocurrents induced by varying light intensity in neurons from Thy1-NpHR (age P13–36; *n* = 6) and Thy1-eNpHR 2.0 (P17 line 4, *n* = 9) mice. **(C)** Both the activation (rise time constant) and deactivation (decay time constant) of photocurrents is slower for NpHR 1.0 than for NpHR 2.0 (mice line 2 and 4 together). Data for Thy1-NpHR mice modified from Zhao et al. (2008). Measured time constants for activation did not take into account slow inactivation of the currents, which should have little effect because activation is much more rapid than inactivation.

To evaluate the efficacy of photoinhibition by NpHR 2.0 vs. NpHR, we compared our results in Thy1-eNpHR2.0 mice to those reported for Thy1-NpHR mice by Zhao et al. (2008). In cortical layer 5 pyramidal cells from Thy1-NpHR mice, illumination induced outward photocurrents (Figure 4A) as described in Zhao et al. (2008). To compare the properties of these photocurrents to those recorded from the same type of neuron expressing eNpHR2.0 (measured in line 2), we first characterized their sensitivity to light by plotting the relationship between light intensity and photocurrent amplitude. For a given light intensity, photocurrents were larger in pyramidal cells expressing eNpHR2.0 than for neurons expressing NpHR (Figure 4B). By fitting these relationships with the Hill equation, we found that the half-maximal light intensity for photocurrent activation was 15 ± 0.2 mW/mm^2^ for NpHR and 0.42 ± 0.02 mW/mm^2^ for eNpHR2.0. Thus, eNpHR2.0 is more light-sensitive than NpHR. In addition, eNpHR2.0 showed significantly faster kinetics, measured as the time constants of photoactivation and deactivation (*p* < 0.001, two tailed *t*-test), compared to NpHR (Figure 4C).

**Figure 5:**
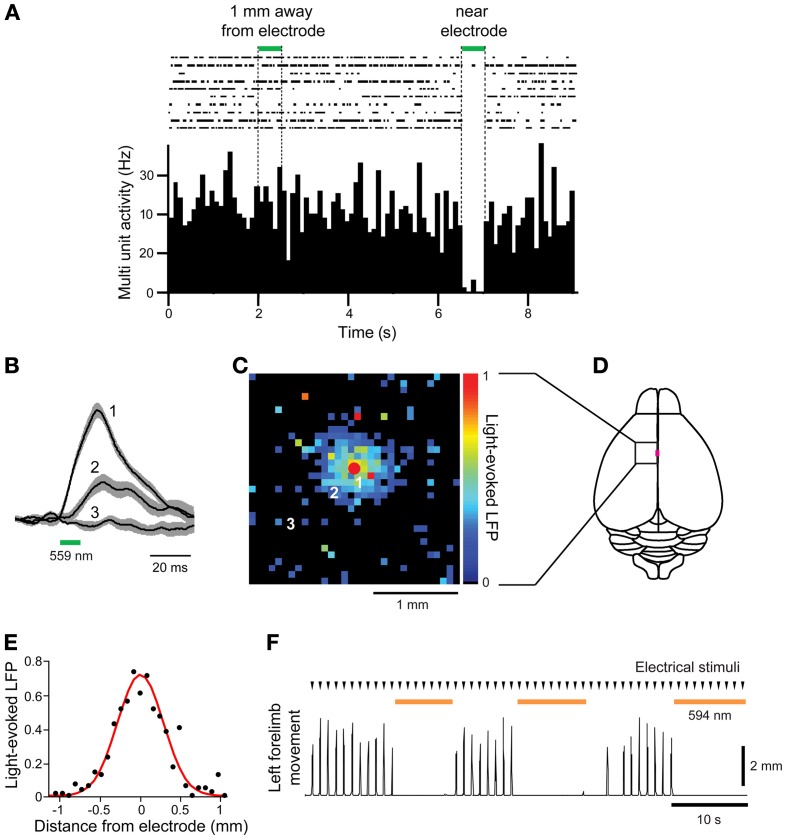
***In vivo* photoinhibition of neuronal activity and limb movement in Thy 1-eNpHR2.0 mice. (A)** Top—Raster display of multiunit activity (MUA) recorded from a single microelectrode in the motor cortex; Bottom—histogram of average MUA frequency. Spot illumination (559 nm) within 0.1 mm of the tip of the recording electrode inhibited spontaneous MUA recorded by the electrode, while illumination with the same spot ~1 mm away from the recording site did not. **(B)** Local field potentials (LFPs) caused by activation of eNpHR2.0, recorded at the location indicated by the red circle in **(C)**, in response to light spots positioned at the numbered locations in **(C)**. **(C)** Map of amplitudes of LFPs evoked when eNpHR2.0 was activated. Each of the 32 × 32 pixels in the map was illuminated (559 nm) and the amplitude of the LFP evoked at each pixel was encoded into the pseudocolor scale shown at right. **(D)** Schematic dorsal view of the cortical surface; boxed region is the photostimulation mapping area and the magenta square denotes the bregma. **(E)** Line scan across the map shown in **(C)** yields the spatial range of photoinhibition, which was 0.65 mm (full-width at half maximum) in this experiment. **(F)** Left forelimb movements were induced by microstimulation at times indicated by arrowheads. Whole-field illumination (594 nm) at the time indicated by orange bars caused a pronounced photoinhibition of forelimb movements.

Finally, we asked whether the Thy1-eNpHR2.0 transgenic mice are suitable for *in vivo* photoinhibition. For this purpose, we illuminated the motor cortex of these mice with green light (559 nm wavelength) while measuring neuronal activity and forelimb movement. First, electrical recordings were used to examine whether illumination inhibited neuronal activity (Figure 5A). Spontaneous multiunit activity (MUA) was recorded from a single electrode that was inserted into the motor cortex. Illumination near the tip of the electrode clearly inhibited spontaneous MUA (Figure 5A), with a rapid recovery of activity afterwards. In contrast, illumination 1 mm away from the recording site had no effect on MUA (Figure 5A).

To examine the spatial range of photoinhibition mediated by eNpHR2.0 *in vivo*, we measured upward LFPs that presumably reflect chloride ion influx into eNpHR 2.0-expressing neurons (Figure 5B). Illumination near the tip of the recording electrode (location 1) induced large LFPs, while illumination at more distant sites produced smaller LFPs (locations 2 and 3). By scanning the position of the light spot, while measuring LFPs, we could make a two-dimensional map of the spatial range of photoinhibition (Figures 5C,D). In five experiments in 2 mice, LFP responses were observed in an area centered over the recording electrode, with responses decreasing away from the recording sites (Figure 5C). The width of the area exhibiting light-induced LFPs (Figure 5E) was 0.65 mm in the experiment shown in Figure 5C, with a mean of 0.66 ± 0.04 mm in all 5 experiments.

We also examined *in vivo* photoinhibition of limb movements induced by intracortical microstimulation in Thy1-eNpHR2.0 mice. Stimulation of the right motor forelimb area in the motor cortex produced movements of the left forelimb. Whole-field illumination of the right cortical surface with orange light (594 nm) clearly inhibited left forelimb movement and movements were restored rapidly once the light was turned off (Figure 5F). However, photoinhibition at a single location with a laser light spot at the same light intensity that inhibited local MUA (Figure 5A) was incapable of inhibiting forelimb movements (data not shown). Thus, to inhibit forelimb movement, the activity of many neurons in a large area (>0.66 × 0.66 mm) must be silenced. These results show that cortical activity and limb movement can be photoinhibited *in vivo* using the Thy1-eNpHR2.0 mouse, indicating that this mouse is an excellent tool for disruption of neural circuit activity *in vivo*.

In summary, photoinhibition of cortical pyramidal cells is more effective in Thy1-eNpHR2.0 transgenic mice in comparison to the original Thy1-NpHR transgenic mice. Thus, the Thy1-eNpHR2.0 mouse provides a better means of using photoinhibition to analyze neuronal circuits.

### Mapping neural circuits with VChR1 transgenic mice

**Figure 6:**
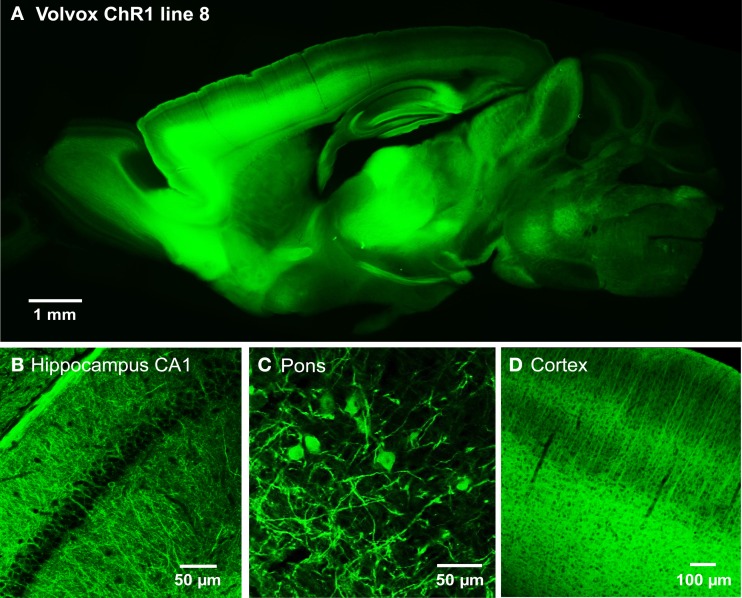
**Expression of VChR1 in transgenic mouse brain. (A)** Sagittal section from a Thy1-VChR1 transgenic mouse brain (line 8, age P29), revealing widespread expression of VChR1 in many brain regions. **(B–D)** Expression of VChR1 in neurons within indicated brain regions.

We next developed the first transgenic mice that express VChR1, a light-gated cation channel that is sensitive to visible light over a very wide range of wavelengths (Zhang et al., 2008). We engineered multiple transgenic mouse lines that drive VChR1-EYFP expression under the Thy1.2 promoter and characterized two of these, termed lines 4 and 8. Both lines showed substantial VChR1 expression in multiple regions of the brain (Figure 6A), such as hippocampus (Figure 6B), pons (Figure 6C), cerebral cortex (Figure 6D), cerebellum, and midbrain. A detailed description of the expression of VChR1 in line 8 is provided below in Table 2. In general, VChR1 seemed to be well-targeted to the plasma membrane, as evident in Figure 6C for example, though there was some intracellular aggregation of VChR1 in neurons within the lateroposterior thalamic nuclei (Table 2 below)

**Figure 7:**
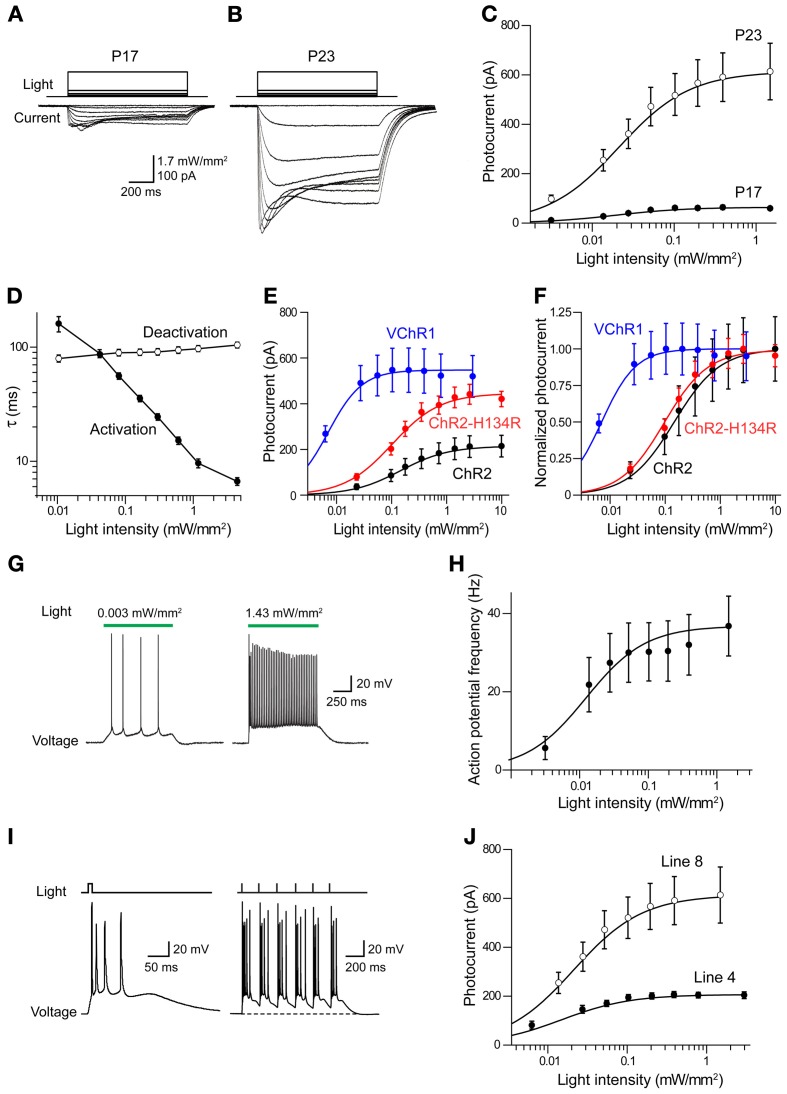
**Photostimulation of cortical pyramidal cells via VChR1. (A)** Illumination (540 nm, 1 s duration) evokes inward photocurrents (bottom) in pyramidal cell in a cortical slice from a P17 Thy1-VChR1 mouse (line 8). The amplitude of the photocurrent varies with the intensity of the light pulse (top). **(B)** Same experiment in a neuron from a P23 mouse elicits larger photocurrents. **(C)** Relationship between photocurrent amplitude and light intensity for P17 (*n* = 4) and P23 (*n* = 8) mice. Smooth curves are fits of Hill equation. **(D)** Dependence of photocurrent activation and deactivation time constants on the intensity of the light pulse. Measured time constants for activation did not take into account slow inactivation of the currents, which should have little effect because activation is much more rapid than inactivation. **(E)** Comparison of relationship between light intensity (480 nm) and absolute photocurrent amplitude for ChR2, ChR2-H134R, and VChR1. **(F)** Comparison of relationship between light intensity (480 nm) and normalized photocurrent amplitude for ChR2, ChR2-H134R, and VChR1. VChR1 requires an order of magnitude lower light level for half activation, even at 480 nm. **(G)** Photostimulation (540 nm) increased action potential frequency in neurons expressing VChR1, with brighter stimuli evoking more action potentials (line 8 mice). **(H)** Relationship between light intensity and number of light-evoked action potentials in slices from P23 mice (line 8, *n* = 8). Curve is fit of the Hill equation. **(I)** Prolonged depolarization associated with VChR1 activation. Left—a brief light flash (5 ms duration; 1.27 mW/mm^2^) caused a prolonged membrane potential depolarization and repetitive action potential firing. Right—Repeated brief light flashes (6 Hz) induced a sustained depolarization and firing of bursts of action potentials. **(J)** Relationship between light intensity and photocurrent amplitude for responses measured in neurons from line 8 (*n* = 8) and line 4 (*n* = 4) mice (both groups age P23).

To determine the ability of VChR1 to photostimulate neurons, electrophysiological recordings were performed in layer 5 pyramidal cells in cortical slices from Thy1-VChR1 line 8 mice. Illumination (540 nm, 1 s duration) produced inward photocurrents (Figures 7A,B). Peak photocurrents increased with age, varying from very small at P17 (Figure 7A) to greater than 500 pA at P23 (Figure 7B). The relationship between light intensity and photocurrent amplitude could be described by the Hill equation at both ages (Figure 7C). In P17 mice, the maximal photocurrent amplitude was 65.0 ± 3.5 pA and half-maximal light intensity was 0.016 ± 0.003 mW/mm^2^. In P23 mice, the maximal photocurrent amplitude was much greater (632 ± 13.1 pA), while the half-maximal light intensity (0.02 ± 0.001 mW/mm^2^) was very similar at both ages. Thus, the level of VChR1 expression increases with age, very similar to what was observed for eNpHR2.0 expression driven by the Thy1.2 promoter in the same neuron type (Figure 3).

The time course of VChR1 activation depended upon the intensity of the light flash, with brighter light stimuli causing more rapid activation (Figure 7B). This effect was quantified by fitting an exponential function to the rising phase of the photocurrents. There was a steep dependence of the time constant of activation (τ) on light intensity, as shown in Figure 7D. In contrast, the time constant for deactivation of the photocurrent, after the end of the light flash, was relatively insensitive to the intensity of the light flash (Figure 7D). The mean time constant for deactivation was 113 ± 2.8 ms (*n* = 9), which was significantly slower than the time constant of 50.7 ± 9.4 ms measured for deactivation of ChR2 in layer 5 pyramidal cells. When VChR1 was activated by 465–495 nm light, the maximum whole cell current induced was 613 ± 256 pA, which was larger than the maximum current recorded in pyramidal cells from mice expressing either ChR2 or ChR2-H134R behind the Thy1 promoter (Figure 7E). The half-maximal intensity was ~0.06 mW/mm^2^ (Figure 7F), which was lower than that measured in pyramidal cells expressing ChR2 (0.14 mW/mm^2^) or ChR2-H134R (0.11 mW/mm^2^), confirming previous indications that VChR1 is more light-sensitive than ChR2 (Berglund et al., 2013).

We characterized the ability of VChR1 to drive action potential firing in layer 5 pyramidal cells in cortical slices made from P23 mice (line 8). During long-duration (1 s) light flashes (540 nm), varying light intensity caused the pyramidal cells to fire at different rates (Figure 7G). The relationship between light intensity and action potential frequency could be described by the Hill equation (Figure 7H), with a maximum action potential frequency of 32.2 ± 1.2 Hz. The half-maximal light intensity of 0.009 ± 0.001 mW/mm^2^ was very similar to what we observed for photocurrents in these neurons (Figures 7C,E). We also applied brief light flashes (5 ms duration) and found that these often induced multiple action potentials (Figure 7I, left). This is quite different from what is observed when photostimulating pyramidal cells in Thy1-ChR2 transgenic mice, where a brief light flash typically elicits one or, at most, two action potentials (Wang et al., 2007). The repetitive firing is due to the prolonged depolarization associated with the slow deactivation kinetics of VChR1 (Figures 7A,B,D). During repetitive photostimulation at a relatively low frequency (6 Hz), this prolonged depolarization summed to yield a sustained depolarizing offset (Figure 7I, right). Thus, while VChR1 is quite effective in depolarizing and firing pyramidal neurons, its slow deactivation kinetics limit the ability to precisely control the timing of action potential firing.

We also measured photocurrents in layer 5 pyramidal cells in cortical slices from Thy1-VChR1 line 4 mice. At age P23, maximal peak photocurrents were 210 ± 3.3 pA, much smaller in comparison to the 632 pA measured for line 8 (Figure 7J). However, the half-maximal light intensity (0.01 ± 0.001 mW/mm^2^) was similar to that measured in line 8. Although these photocurrents could induce sufficient depolarization to fire action potentials (data not shown), layer 5 pyramidal cells were more readily photostimulated in line 8 than in line 4.

**Figure 8:**
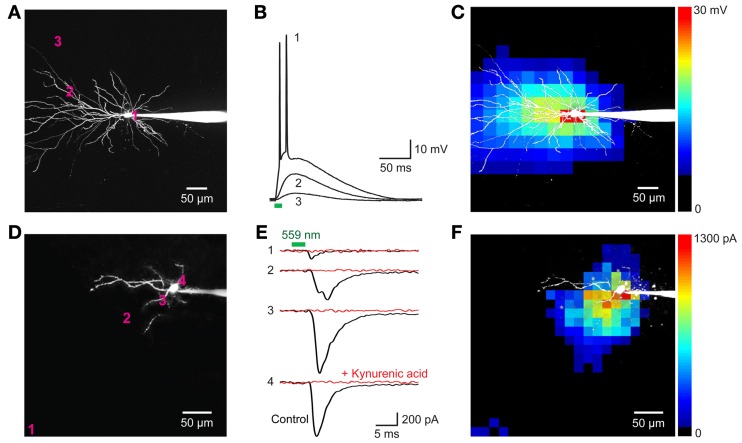
**Neuronal circuit mapping with Thy1-VChR1 transgenic mice. (A–C)** Mapping the light sensitivity of a VChR1-expressing CA1 pyramidal neuron in a hippocampal slice (line 8). **(A)** 2-photon image of a pyramidal neuron filled with Alexa 594 dye; patch pipette is to the right. The numbers on the cell image indicate locations where the responses shown in *B* were evoked. **(B)** Changes in membrane potential evoked by laser light spots (559 nm; 27 μW) positioned at the sites indicated in **(A)**. Only illumination near the cell body evoked action potentials (trace 1). Bar below the traces indicates the time of illumination (8 ms). **(C)** Scanning the light spot across the specimen revealed locations where light-induced depolarizations were evoked; pseudocolor scale at right indicates the amplitude of these responses. Red pixels reflect regions where action potentials were evoked. **(D–F)** Mapping of local excitatory circuits within the nucleus reticularis tegmenti pontis (NRTP; line 8). **(D)** Structure of NRTP neuron filled with Alexa 594 dye. The numbers indicate locations where photostimulation evoked the synaptic responses shown in **(E)**. **(E)** Light-induced postsynaptic currents (holding potential = −70 mV), detected when a laser light spot (559 nm, 280 μW) was positioned at the locations indicated in **(F)**. Black traces indicate responses recorded in control conditions and red traces indicate responses measured after treatment with kynurenic acid (2 mM). Bars above traces indicate time of illumination. **(F)** Map of locations where light evoked EPSCs; the magnitude of these currents is indicated by the pseudocolor scale at right.

We next determined whether VChR1 could be used for functional mapping of neuronal circuits in various brain regions. For this purpose, we used small laser light spots (594 nm; 8 ms duration; ≈1.1 μm diameter in the focal plane) to locally stimulate presynaptic neurons expressing VChR1. To determine how spatially precise the photostimulation was, we mapped the sensitivity of individual VChR1-expressing neurons to scanned light spots (Wang et al., 2007; Schoenenberger et al., 2008; Lewis et al., 2009; Kim et al., in revision). To accommodate the slow deactivation kinetics of VChR1, the time interval between photostimuli was set at 500 ms to allow time for responses to fully recover between stimuli. Figures 8A–C indicates one example of a map of the light sensitivity of a patch-clamped hippocampal CA1 pyramidal neuron expressing VChR1 (line 8 mouse). This neuron was filled with a fluorescent dye (Alexa 594) to visualize its structure (Figure 8A). When the laser light spot was scanned in a two-dimensional array through the brain slice, depolarizing responses could be elicited when the light spot was positioned over virtually any region of the cell (Figure 8B). However, responses were largest when the light spot was located in the immediate vicinity of the neuronal cell body (trace 1 in Figure 8B). The higher sensitivity of the somatic region presumably arises from the relatively large surface area exposed to the light beam in this region. If the intensity of the light spot was adjusted appropriately, action potentials were evoked only when the light spot was over the cell body (red pixels in Figure 8C). In this case, only 1 or 2 action potentials were evoked, in contrast to the more robust action potential trains observed when the entire neuron was illuminated simultaneously (for example, Figure 7I, left). This presumably occurs because fewer VChR1 molecules are activated by the focal laser spot. As expected these direct responses were unchanged in the presence of kynurenic acid (2 mM), a blocker of glutamatergic neurotransmission.

The ability to selectively activate neurons when the light beam is over (or near) their somata makes it possible to map circuit connectivity (Petreanu et al., 2007; Wang et al., 2007; Mao et al., 2011; Kim et al., in revision). In such experiments, the laser spot is scanned to focally photostimulate small numbers of presynaptic neurons expressing a light-activated channel, VChR1 in this case, while postsynaptic responses are detected in non-expressing neurons. Locations where postsynaptic responses are evoked then indicate the location of presynaptic input neurons.

Figures 8D–F provides an example of such a circuit mapping experiment, in this case visualizing local excitatory microcircuitry within the nucleus reticularis tegmenti pontis (NRTP) of the pons. A recording was made from a NRTP neuron (Figure 8D) that did not express VChR1, evident as the absence of photocurrents in response to light after treating the slice with kynurenic acid to block excitatory synaptic transmission (Figure 8E, red traces). Illumination with a laser spot (230 μW) elicited inward currents in the NRTP neuron when the spot was positioned at various locations within the slice (Figure 8E, black traces). Several criteria demonstrated that these responses were excitatory postsynaptic currents (EPSCs) rather than photocurrents resulting from activation of VChR1. First, they appeared several milliseconds after the light stimulus (green bar in Figure 8E), whereas direct photoresponses occurred with negligible delay (Figures 7, 8B). Second, the responses sometimes had multiple peaks (for example, trace 2 in Figure 8E), presumably because of activation of multiple presynaptic neurons (or repetitive firing in a single presynaptic cell), whereas the direct photocurrents developed smoothly and did not have multiple peaks. Third, the responses were eliminated by kynurenic acid, a glutamate receptor antagonist (Figure 8E, red traces).

In the example shown in Figure 8E, illumination at locations near the NRTP neuron, such as sites 2, 3, and 4, elicited EPSCs. These sites indicate the location of the somata/proximal dendrites of VChR1-positive presynaptic glutamatergic inputs. By scanning the light spot in two dimensions, it was possible to map the spatial distribution of all VChR1-positive neurons that provide synaptic input to this NRTP neuron. The resulting map is shown in Figure 8F, with the amplitude of the evoked EPSCs encoded into the pseudocolor scale shown on the right. Such local excitatory microcircuitry was observed in a total of 3 replicates in this preparation. In this experiment, there was an additional remote input coming from the distal direction, evident as a cluster of responsive pixels in the vicinity of site 1 (lower left corner of Figure 8F). Similar longer-range excitatory inputs were observed in 12 out of 20 experiments carried out in the same manner. Thus, photostimulation of VChR1-expressing NRTP neurons can define the spatial organization of circuits formed between these neurons and their postsynaptic partners. More generally, these results indicate that Thy1-VChR1 transgenic mice can be useful for mapping the spatial organization of both local and longer-range synaptic circuits.

### Fluorescent tagging of ChR2 is fraught with peril: limits on expression and trafficking of tagged ChR2

**Figure 9:**
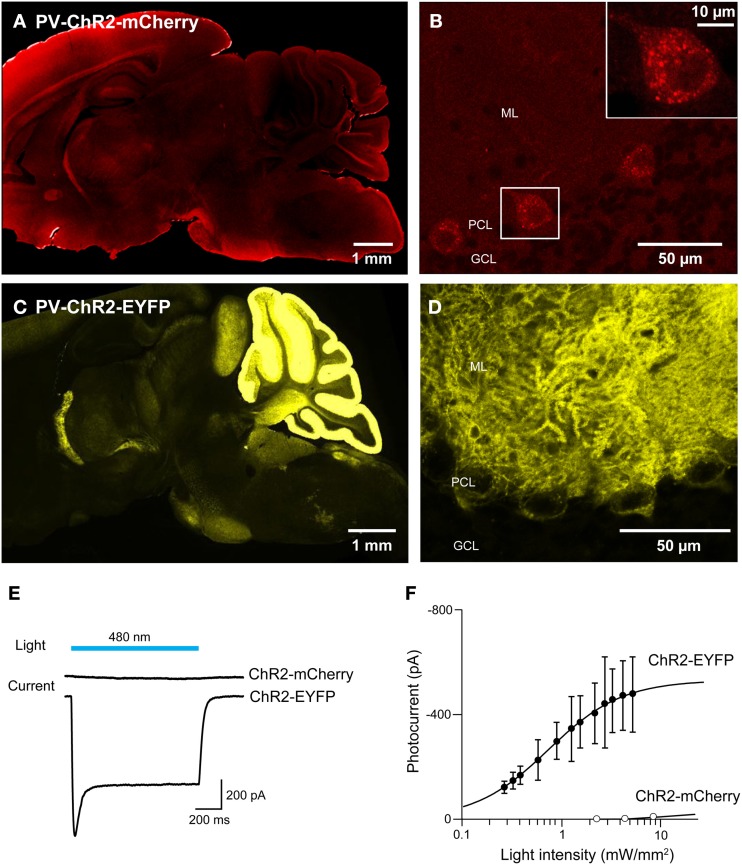
**Comparison of ChR2 tagged with mCherry and EYFP. (A,B)** Sagittal sections from the whole brain **(A)** and cerebellum **(B)** of a PV-ChR2-mCherry mouse. **(C,D)** Images of sagittal sections from the whole brain **(C)** and cerebellum **(D)** of a PV-ChR2-EYFP mouse. **(E)** Peak photocurrents (lower traces) induced by light (480 nm; top) in Purkinje cells from ChR2-mCherry (middle) or ChR2-EYFP mice (bottom). Light intensity was 4.5 mW/mm^2^ for ChR2-mCherry and 5.3 mW/mm^2^ for EYFP. **(F)** Relationship between light intensity and photocurrent amplitude for the two lines (*n* = 8 cells for ChR2-mCherry and *n* = 4 for ChR2-EYFP). Error bars denote sem.

For experiments employing multiple optogenetic probes, or combining optogenetic manipulation of neurons with fluorescent imaging, it is essential to have probes with spectrally-separable fluorescent tags. For this reason, we tagged ChR2 with fluorescent proteins other than EYFP. We first created BAC transgenic mice in which ChR2 was tagged with the red fluorescent protein, mCherry (Shaner et al., 2004) and expressed under the parvalbumin promoter (PV-ChR2-mCherry; Seto-Ohshima et al., 1989; Kawaguchi, 1995; Maccaferri et al., 2000). To assess the effects of the mCherry tag, we also created another new mouse line where the parvalbumin promoter was used to drive expression of ChR2 tagged with EYFP (PV-ChR2-EYFP). Out of several transgenic founders, we found ChR2-mCherry expression in the brain in only one line. In this line, there was diffuse and dim expression of ChR2-mCherry in the hippocampus, the neocortex, and the cerebellar cortex (Figure 9A). We concentrated on the cerebellum, where fluorescent Purkinje cells were observed (Figure 9B) and ChR2-mCherry seen predominantly in cytoplasmic puncta. In contrast, ChR2-EFYP expression was observed in many lines of PV-ChR2-EYFP mice. In the line selected for characterization, ChR2 was predominantly expressed in the molecular and Purkinje cell layers of the cerebellum (Figure 9C) and exhibited the expected cell surface labeling of Purkinje cell somata by ChR2-EYFP (Figure 9D).

To characterize activation of ChR2 in these two mouse lines, whole-cell patch-clamp recordings were made from fluorescent Purkinje cells in acute cerebellar slices. Figure 9E shows representative traces of photocurrents recorded in Purkinje cells from the two lines of mice. Illumination by a light pulse (480 nm) caused no detectable photocurrent in cells from PV-ChR2-mCherry mice (Figure 9E, center), even with very bright light pulses. In contrast, even dim light flashes caused robust photocurrents in Purkinje cells from PV-ChR2-EYFP mice (Figure 9E, bottom). For Purkinje cells from PV-ChR2-EYFP mice, the relationship between light intensity and photocurrent amplitude could be described by the Hill equation (Figure 9F; Wang et al., 2007), with a maximal current of 530 ± 9 ṗA and half-maximal current observed at 0.750 ± 0.028 mW/mm^2^ (*n* = 4). The lack of measurable photocurrents made it impossible to fit the relationship for Purkinje cells from PV-ChR2-mCherry mice (*n* = 8). In summary, intracellular sequestration of ChR2-mCherry apparently is responsible for poor surface expression and negligible photocurrents in PV-ChR2-mCherry mice. In contrast, in PV-ChR2-EYFP mice the ChR2-EYFP was properly delivered to the cell membrane and yielded robust photocurrents upon light stimulation. Because we used the same promoter and the same optogenetic actuator in the two lines, the simplest interpretation of our results is that chronic expression of the mCherry tag causes ChR2 to aggregate in transgenic mouse brains.

**Figure 10:**
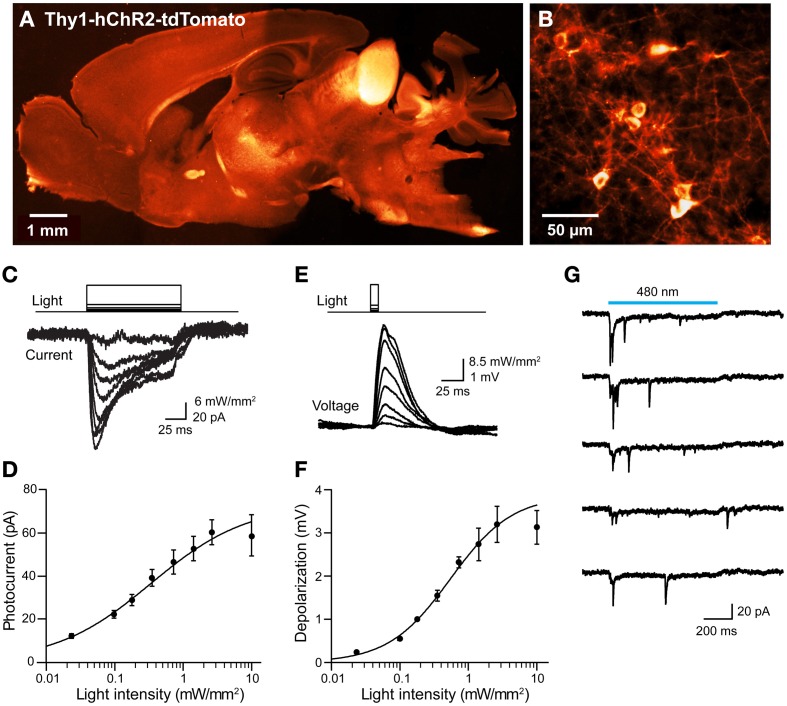
**Mild photostimulation with ChR2 tagged with tdTomato. (A)** Sagittal brain section from a Thy1-hChR2-tdTomato mouse. **(B)** Higher-resolution image of cortical pyramidal cells. **(C)** Photocurrents (bottom) evoked by light pulses (top, 480 nm, 100 ms duration) in a pyramidal cell from a cortical slice from ChR2-tdTomato mouse. **(D)** Relationship between light intensity and photocurrent amplitude in cortical neurons (*n* = 7; means ± sem). Smooth curve is fit of Hill equation. **(E)** Changes in membrane potential evoked by brief (10 ms, 480 nm) light flashes in a cortical pyramidal neuron. Depolarizations typically were too small to evoke action potentials. **(F)** Relationship between light intensity and membrane potential depolarization in cortical neurons (*n* = 2; means ± sem). Smooth curve is fit of Hill equation. **(G)** Synaptic currents evoked in a cortical pyramidal neuron that expressed minimal ChR2-tdTomato. When the slice was illuminated with 480 nm light, multiple EPSCs were elicited during the light flash (750 ms duration). Traces indicate responses to five repeated exposures to the same light stimulus.

We next considered the orange fluorescent protein, tdTomato (Shaner et al., 2004), as an alternative. We created transgenic mice where tdTomato-ChR2 expression was driven by the Thy1 promoter. We imaged tdTomato fluorescence in parasagittal sections and found diffuse signal in many brain regions, with brighter labeling in the thalamus, midbrain, and brainstem, as well as some fiber tracts within the striatum and corpus callosum (Figure 10A). Higher-resolution imaging revealed patchy labeling in the cerebral cortex (Figure 10B), as well as labeling in the granular cell layer of the cerebellum. Imaging of live cortical slices revealed a range of tdTomato fluorescence in pyramidal cell bodies, with the major neuronal processes often visible in brightly labeled cells. The most fluorescent pyramidal cells quickly exhibited large leakage current after establishing the whole-cell recording configuration and subsequently died. Thus, it appears that long-term high expression of ChR2-tdTomato somehow causes pyramidal cells to become very fragile during patch clamp recordings. We next recorded from pyramidal cells exhibiting dimmer fluorescence and saw relatively small photocurrents, on the order of tens of pA in response to light pulses (480 nm; Figure 10C). The relationship between light intensity and photocurrent amplitude (Figure 10D) could be described by the Hill equation, with a maximal current of 73 ± 43 pA and half-maximal luminance of 0.34 ± 0.7 mW/mm^2^ (*n* = 7), several-fold smaller than is observed for ChR2 (or VChR1) tagged with EYFP. Similarly, illumination caused small, subthreshold depolarizations (Figure 10E), with a maximal voltage response, calculated from fits of the Hill equation (Figure 10F), of 3.9 ± 1.0 mV, and half-maximal luminance of 0.54 ± 0.28 mW/mm^2^ (mean ± 1 SD; *n* = 2). Though we were seldom able to evoke action potential firing in individual pyramidal cells in response to illumination, it was possible to observe light-evoked EPSCs (Figure 10G). This presumably is due to photostimulation of neighboring pyramidal cells that express sufficient ChR2-tdTomato to generate action potentials that elicit excitatory synaptic transmission.

In summary, tdTomato proved better than mCherry as a fluorescent tag for ChR2: ChR2-tdTomato was expressed on the plasma membrane and yielded modest photocurrents in response to light. However, because cells with long-term expression of high levels of ChR2-tdTomato were fragile and could not be used for electrophysiological analyses, chronic expression of tdTomato-ChR2 in transgenic mice apparently is not optimal for optogenetic control of neuronal activity. Thus, for tagging ChR2 in transgenic mice, we advise the use of EYFP, which has no discernible adverse effects on neuronal ChR2 expression after chronic expression. Alternatively, two-color labeling could be achieved by acute expression (e.g., viral) of a red-tagged opsin in any line chronically expressing EYFP-tagged opsins.

### Genetic strategies for optogenetic control of cerebellar purkinje cells

Because we are very interested in optogenetic control of cerebellar Purkinje cells, we compared three alternative strategies for expressing ChR2 specifically in Purkinje cells. The parvalbumin (PV) promoter was employed to drive expression of EYFP-tagged ChR2 in a variety of neurons, including Purkinje cells, in two BAC transgenic mice lines: one which we created expressing ChR2, called PV, and another, termed line 15, that expressed ChR2-H134R. The PV line was briefly introduced above (Figure 9), while the properties of line 15 have previously been described by Zhao et al. (2011). Another promoter, PCP2 (Purkinje cell protein 2), was used to target ChR2-H134R expression more exclusively to Purkinje neurons. This third mouse line (termed PCP2) was obtained by crossing a transgenic mouse that expresses Cre recombinase under control of the PCP2 promoter (Barski et al., 2000; Zhang et al., 2004) with another transgenic mouse expressing ChR2-H134R behind a floxed stop cassette (Madisen et al., 2010, 2012). When these two mice are mated, Cre removes the stop signal and ChR2-H134R is expressed.

**Figure 11:**
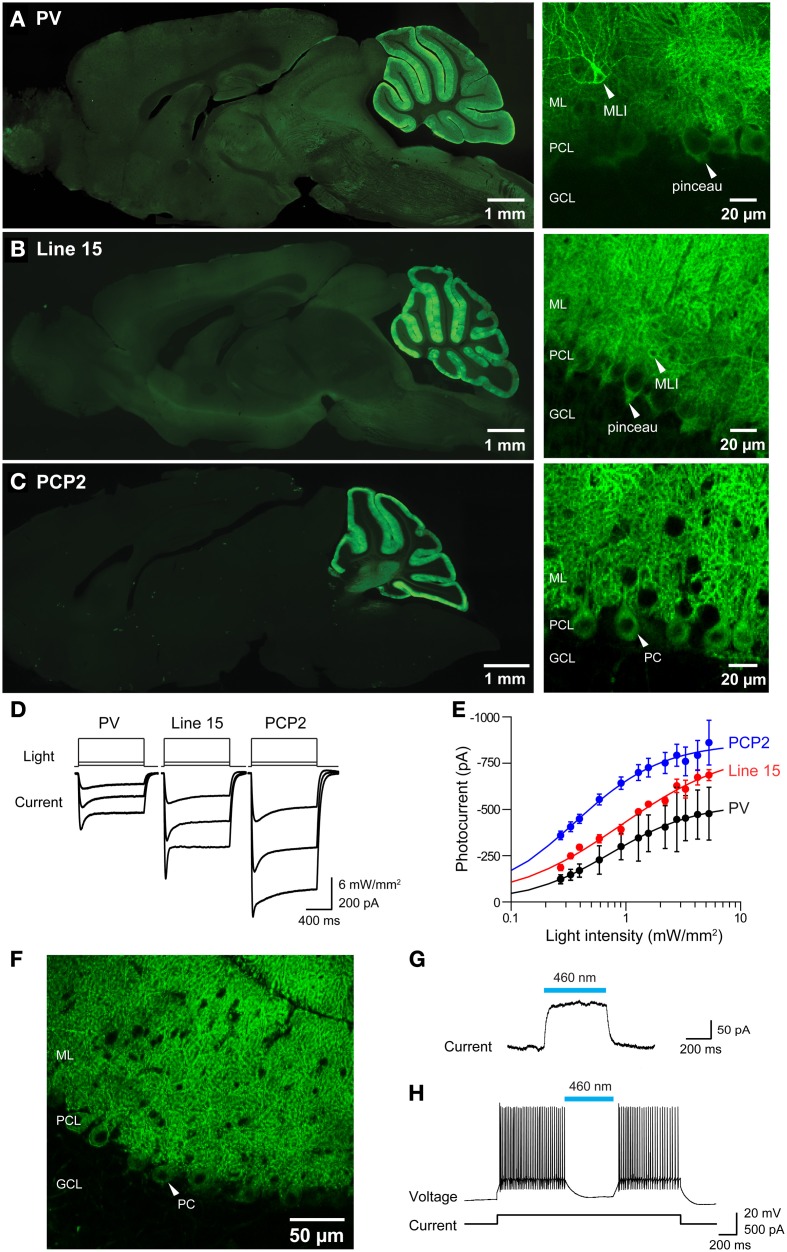
**Photostimulation of Purkinje cells in 3 lines of ChR2 transgenic mice. (A–C)** Images of ChR2-EYFP fluorescence in sagittal sections from brains of PV, Line 15 and PCP2 mice. Left—whole-brain images. Right: high magnification images showing ChR2-EYFP expression in cerebellar molecular layer interneurons (MLI), pinceau terminals and Purkinje neurons (PC). ML, molecular layer; PCL, Purkinje cell layer; GCL, granular cell layer. **(D)** Photocurrents (bottom) elicited by light pulses (top) in Purkinje cells in cerebellar slices from the 3 mouse lines (for information see Table 1). **(E)** Relationship between photocurrent amplitude and light intensity; curves are fits of the Hill equation. (PV, *n* = 4; Line 15, *n* = 5; PCP2, *n* = 11). **(F)** Expression of Arch-EGFP in cerebellar Purkinje cells. **(G)** Outward photocurrent induced in a Purkinje cell by illumination (460 nm; 29.6 mW/mm^2^). Holding potential was −60 mV. **(H)** Photoinhibition of Purkinje cell activity by blue light (460 nm; 144 mW/mm^2^). Same cell as in **(G)**.

Histological analyses revealed prominent ChR2 expression in the cerebellum of all three mouse lines (Figures 11A–C). Within the cerebellar cortex, expression of ChR2 was abundant in Purkinje neurons in mice from all three lines and was most pronounced in the molecular layer (ML) of the cerebellar cortex, where the dendritic arbors of Purkinje neurons reside (Figures 11A–C, right). Expression within Purkinje cell somata was apparent in the Purkinje cell layer (PCL) and fluorescent pinceau terminals were evident around the base of Purkinje cells (Figures 11A,B, right) in PV and line 15 mice, indicating ChR2 expression in the molecular layer interneurons (MLI). In these two lines, fluorescent MLI somata could be seen occasionally and with variable intensities. In contrast, expression was not observed in MLI of PCP2 mice and ChR2 expression was limited to Purkinje cells in these mice (Figure 11C, right). ChR2 was also observed in the deep cerebellar nuclei (DCN), at least partially due to ChR2 expression in Purkinje cell axons. A detailed description of the expression of ChR2 in these lines is provided in Table 2 below.

To determine whether the ChR2 expressed in these mice was functional, we measured photocurrents in Purkinje cells in cerebellar slices. Wide-field illumination (0.016 mm^2^ area, 460 nm) elicited robust photocurrents that slowly inactivated (Figure 11D). The amplitude of these photocurrents varied according to the intensity of the light pulse. At a given light intensity, photocurrents were largest for PCP2 and smallest for PV (Figure 11E). The relationship between peak photocurrent amplitude and light intensity was well-described by the Hill equation for mice from all 3 lines (Figure 11E). This analysis indicates that maximum photocurrent amplitude varied for Purkinje cells in the three lines, with largest photocurrents evoked by a given light intensity in PCP2 mice: maximum of 860 ± 24 pA and a half-maximal luminance of 0.36 ± 0.02 mW/mm^2^ (*n* = 7). The maximum photocurrent in line 15 Purkinje cells (833 ± 67 pA) was not significantly different, with the relationship between light intensity and photocurrent amplitude having a somewhat higher half-maximal luminance of 0.91 ± 0.20 mW/mm^2^ (*n* = 4). Purkinje cells in PV mice had the lowest maximal photocurrent of 533 ± 18 pA and a half-maximal luminance of 0.74 ± 0.08 mW/mm^2^ (*n* = 4). The larger photocurrents in line 15 mice compared to those in the PV mice may be due to the H134R gain-of function substitution in line 15 (Nagel et al., 2005; Gradinaru et al., 2007). Because the PCP2 mice also express ChR2 with the H134R mutation, the strong promoter associated with the floxed ChR2 (Madisen et al., 2010, 2012) may also enhance photocurrents by driving strong expression of ChR2. Demonstrating this, crossing the PCP2-Cre mice with floxed Arch mice (Madisen et al., 2012) yielded strong expression of this proton pump exclusively in Purkinje cells (Figure 11F). Illumination (460 nm) caused outward photocurrents (Figure 11G) that powerfully photoinhibited Purkinje cells from firing action potentials (Figure 11H).

**Figure 12:**
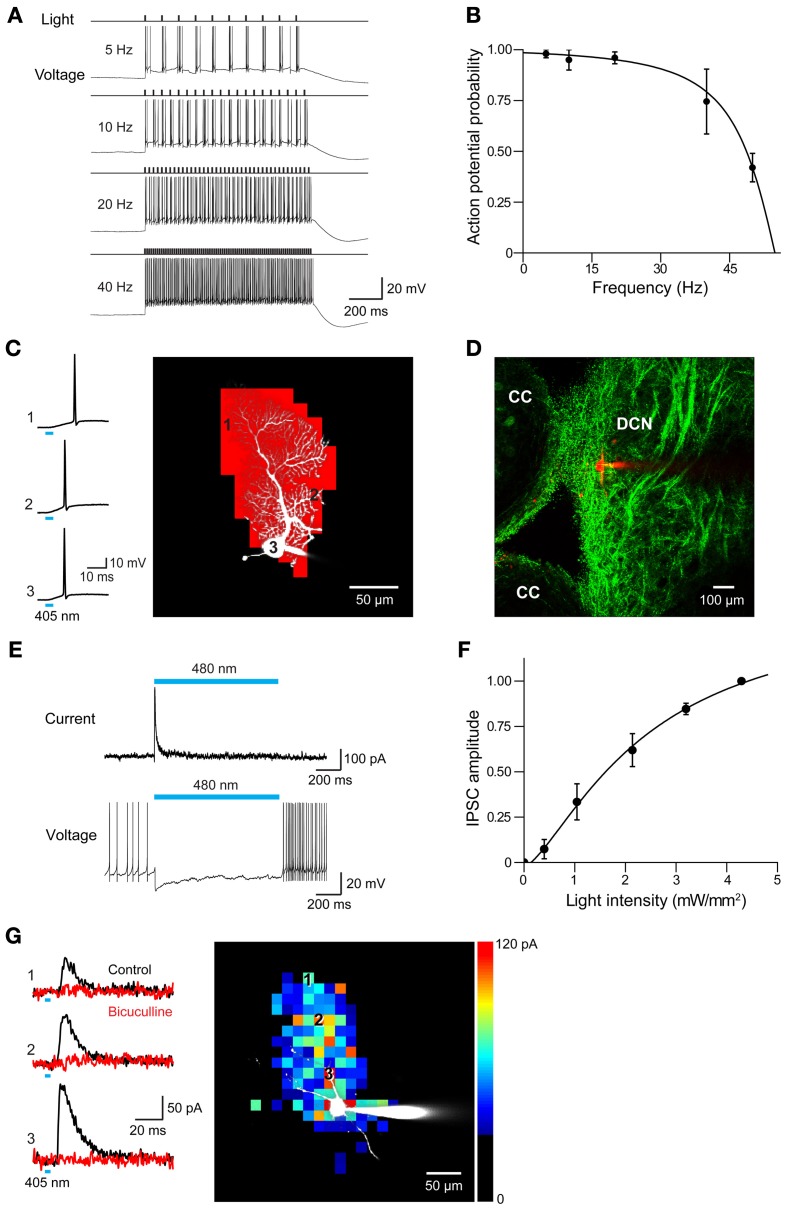
**Photostimulation of Purkinje cells allows mapping of DCN inhibitory circuits. (A)** Action potentials evoked in a Purkinje cell from PV mouse line by repetitive light flashes (480 nm, 11.7 mW/mm^2^, 10 ms duration) applied at various frequencies. **(B)** Relationship between photostimulus frequency and probability of evoking action potentials in a Purkinje cell. Smooth curve is the fit of a Lorentzian function with a cut-off frequency of 47 Hz (*n* = 3). **(C)** Mapping the light sensitivity of a ChR2-expressing Purkinje cell in a cerebellar slice (PV line). Left—Action potentials evoked by brief (405 nm, 0.27 mW, 4 ms duration) laser light spots. Numbers represent locations indicated by the numbered pixels in the image at right. Bar below the traces indicates the time of illumination (8 ms). Right—Scanning the light spot across the slice revealed locations (red pixels) where light evoked action potentials in the Purkinje cell. **(D)** Image showing ChR2-YFP expression in DCN and surrounding cerebellar cortex (CC). Red shows image of dye-filled DCN neuron. **(E)** Light-induced IPSC (upper) and IPSP (lower) measured in a DCN neuron in response to 2 s illumination (480 nm, 11.7 mW/mm^2^) of a cerebellar slice. **(F)** Relationship between light intensity (405 nm, 6 ms duration) and IPSC amplitude measured in DCN neurons (*n* = 6). Curve is a fit of the Hill equation. **(G)** Optogenetic mapping of inhibitory inputs to a DCN neuron. The amplitude of light-evoked IPSCs (left, black traces) recorded at the indicated locations (image) was mapped in the pseudocolor scale shown at right. Responses were blocked by bicuculline (red traces), confirming that they are IPSCs. Laser pulses were 405 nm, 0.48 mW, and 4 ms duration.

In all 3 ChR2 lines, photostimulation evoked action potentials in Purkinje cells. Examples of the efficacy of photostimulation in the PV line, which exhibited the smallest photocurrents of the 3, are shown in Figure 12A. In cerebellar slices from these mice, action potentials could be reliably evoked by light pulses (10 ms duration, 480 nm) at frequencies up to 40 Hz (Figure 12B), with the highest frequency response obtained with the highest illumination intensities. Extra spikes were evident during low-frequency photostimulation; this may be due to the slow deactivation kinetics of the ChR2 (Gunaydin et al., 2010; Zhao et al., 2011), as well as the intrinsic excitability properties of Purkinje cells (Llinas and Sugimori, 1980; Chang et al., 1993). Using the same procedure illustrated in Figures 8A–C, we mapped the sensitivity of individual Purkinje cells to scanned light spots (4 ms duration; 405 nm). In the response map shown in Figure 12C, red pixels indicate locations where light spots evoked action potentials. In this case, focal photostimuli evoked action potentials when positioned over the Purkinje soma or its dendrites. In adjacent areas, photostimuli elicited subthreshold depolarizations. Different from what is found for most other central neurons expressing ChR2 (e.g., Figure 8C, as well as Wang et al., 2007; Kim et al., in revision), where somata are typically most sensitive to light, Purkinje cell dendrites were most sensitive to light so that action potentials were evoked at lower light intensities than those required when the light spot was positioned over the cell body (data not shown; see Augustine et al., 2013). This presumably is due to the unique structure of Purkinje cell dendrites, which causes a relatively large membrane surface area to be located within the light spot.

To determine whether these mice are useful for mapping Purkinje cell circuits, we asked whether photostimulation of Purkinje cells elicits responses in postsynaptic neurons. For this purpose, we recorded from neurons in DCN in sagittal slices from PV mice. ChR2 expression in DCN of these mice was observed in axonal structures and dim expression occasionally was also observed in DCN cell bodies (Figure 12D). This is consistent with previous descriptions of PV expression in the cerebellum (Bäurle et al., 1998; Schwaller et al., 2002). Light spots (0.49 mm diameter, 0.76 mm^2^ area) were centered over the DCN and covered the DCN, as well as part of the cerebellar cortex. Illumination (480 nm, 2 s duration) evoked inhibitory postsynaptic currents (IPSCs) in DCN neurons (Figure 12E, top). Because illumination never evoked action potentials in DCN neurons (*n* = 47; data not shown), these responses presumably are due to photostimulation of the axons of presynaptic Purkinje cells. Such axonal photostimulation was enabled by the use of a large light spot. The inhibitory postsynaptic potentials (IPSPs) evoked by photostimulation attenuated spontaneous firing in DCN neurons (Figure 12E, bottom). Varying photostimulus intensity altered the size but not the time to peak of the light-evoked IPSC (Figure 12F). Together these results reflect recruitment of a variable number of Purkinje cell inputs and is consistent with previous work demonstrating the convergence of a large number of Purkinje cell inputs onto DCN neurons (Anchisi et al., 2001; Gauck and Jaeger, 2003; Person and Raman, 2012).

To map the spatial organization of these Purkinje cell inputs onto DCN neurons, we scanned a small but bright laser spot (6 ms duration; 405 nm) while recording IPSCs from postsynaptic DCN neurons. Photostimulation evoked IPSCs at many locations (Figure 12G, left); blockade by bicuculline (10 μM) confirmed that these responses were indeed IPSCs. The spatial map of these response locations (Figure 12G, right) revealed that Purkinje cell inputs are spread over a wide area within the dorsal-ventral axis. It is likely that this represents innervation by a bundle of Purkinje cell axons photostimulated by the bright light spot, with these axons diverging out of the slice at the top of the map.

In summary, all 3 mouse lines allow photostimulation of Purkinje cells. This capability enables mapping of Purkinje cell circuitry, among many other applications. Because the PCP2 mice exhibit highest ChR2 expression in Purkinje cells, and negligible expression in MLI, these mice will be the preferred option except for applications where the slower deactivation kinetics of the H134R mutation might be limiting.

### Photostimulation mapping of cortical interneuron circuits

**Figure 13:**
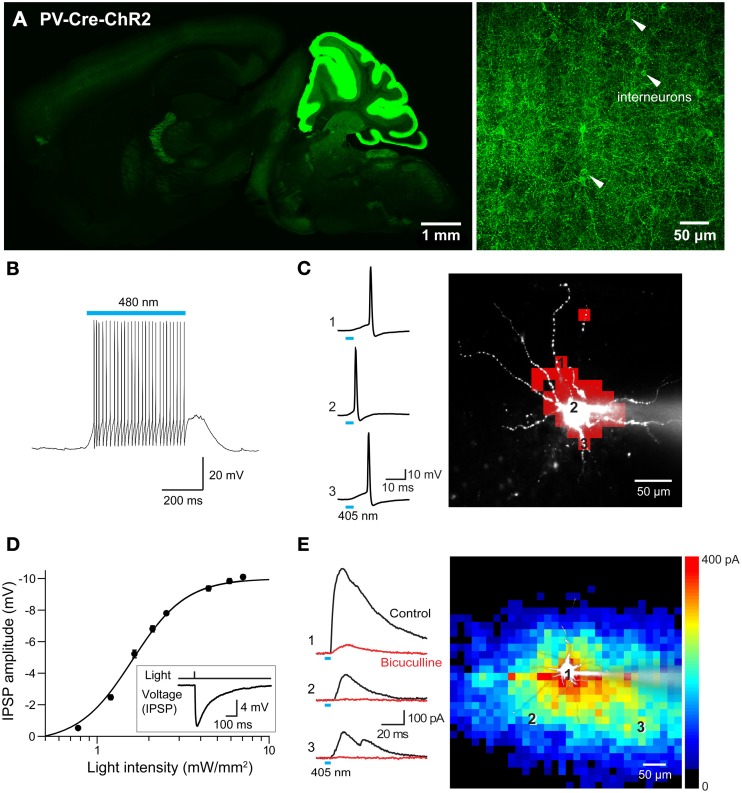
**Mapping interneuronal circuits using floxed ChR2 mice. (A)** ChR2-EYFP expression in a sagittal section from brain of a PV-Cre × double-floxed stop ChR2 transgenic mouse. Left—low magnification image of whole brain. Right—high magnification image of somatosensory cortex showing interneurons expressing ChR2-EYFP in their plasma membrane (white arrowheads). **(B)** Photostimulation (480 nm, 500 ms duration) evoked action potentials in an interneuron expressing ChR2. **(C)** Mapping the light sensitivity of a ChR2-expressing interneuron in a cortical slice. Left—Action potentials evoked by brief (405 nm, 0.54 mW, 4 ms duration) laser light spots. Numbers represent locations indicated by the numbered pixels in the image at right. Bar below the traces indicates the time of illumination. Right—Scanning the light spot across the slice revealed locations (red pixels) where light evoked action potentials in the interneuron. **(D)** Relationship between light intensity and amplitude of IPSPs evoked in a cortical pyramidal neuron in response to wide-field (460 nm) photostimulation of presynaptic interneurons. Curve is fit of the Hill equation. Inset shows IPSP evoked in a cortical pyramidal neuron evoked by 7.1 mW/mm^2^ photostimulus. **(E)** Optogenetic mapping of inhibitory inputs to a cortical pyramidal cell. The amplitude of light-evoked IPSCs (left, black traces) recorded at the indicated locations (image) was mapped in the pseudocolor scale shown at right. Responses were blocked by bicuculline (red traces), confirming that they are IPSCs. Laser pulses were 405 nm, 0.48 mW, and 4 ms duration.

Use of the Cre/lox system to regulate ChR2 expression in transgenic mice opens up many more opportunities for optogenetic control of neurons (Madisen et al., 2012). For example, mating these mice with another mouse line expressing Cre recombinase under the control of the PV promoter (Hippenmeyer et al., 2005) provides an alternative to the PV and line 15 BAC transgenic mice described above. Histological analyses of mice derived from this cross revealed robust ChR2-H134R expression throughout the ML of the cerebellum (Figure 13A, left). Moderate expression could be detected in the reticular thalamic nucleus, inferior colliculus, lateral leminiscus, brainstem, and cerebral cortex (Table 2 below). High-magnification images of cortex revealed expression in presumed PV-interneurons within layers 4–5 (Figure 13A, right) but not in cortical pyramidal neurons.

To extend previous optogenetic analyses of cortical interneurons (Katzel et al., 2011; Atallah et al., 2012; Lee et al., 2012; Wilson et al., 2012), we used laser-scanning photostimulation to map the connections between PV-interneurons and pyramidal neurons. Action potential trains were evoked in the interneurons in response to wide-field illumination (0.56 mm diameter, 500 ms duration, 470–495 nm) of cortical slices (Figure 13B). Adjustment of laser intensity established conditions where action potentials were evoked only when scanned laser spots (405 nm, 4 ms duration) were positioned over the soma or proximal dendrites of these PV-interneurons (Figure 13C).

Photostimulation of PV-interneurons activated inhibitory circuits within the cortex. This was examined via recordings from layer 5 pyramidal cells within the somatosensory barrel cortex. Brief light flashes (4 ms duration, 460 nm) did not cause depolarizations or inward photocurrents, providing further indications that pyramidal cells in these mice do not express ChR2. However, the light flashes did evoke hyperpolarizing IPSPs in these cells (Figure 13D inset). These IPSPs were larger in amplitude with brighter light flashes (Figure 13D), due to recruitment of increasing numbers of presynaptic interneurons. By scanning a small laser spot, while recording from the layer 5 pyramidal cells, it was possible to map the spatial organization of PV interneuron inputs to these pyramidal cells. The example shown in Figure 13E illustrates the relatively wide-ranging input provided to pyramidal cells by PV interneurons. This is similar to input maps obtained by stimulating all major subtypes of *Gad2*-expressing interneurons (Katzel et al., 2011). This map differs in shape from previously published maps of pyramidal cell-pyramidal cell connectivity in the cortex (Wang et al., 2007). It is also apparent that IPSC rise time varied according to the location of the light spot, with locations close to the pyramidal cell body evoking faster IPSCs than those evoked in more distal locations (Figure 13E, left). This could in part be due to dendritic passive filtering of the signals (Magee, 2000) or could reflect a difference in the kinetics of synaptic transmission associated with different presynaptic interneurons (Markram et al., 2004).

In summary, these results indicate that the transgenic mice with ChR2 expression controlled by a floxed stop cassette are very useful tools for mapping the circuits in which presynaptic cortical interneurons (in this case, PV-expressing interneurons) participate, confirming and extending the conclusions of Madisen et al. (2012). More generally, by mating these mice to other Cre driver lines it should be possible to target virtually any type of presynaptic neuron for such studies.

## Discussion

The growing variety of optogenetic actuators, and transgenic mouse lines expressing these optogenetic probes in neurons, provide increasingly valuable opportunities to investigate circuit function in the mouse brain (Mancuso et al., 2011; Yizhar et al., 2011; Zhang et al., 2011; Zhao et al., 2011; Madisen et al., 2012). Here we have characterized several new transgenic mice that reflect various approaches for optogenetic control of neurons and expand the capabilities of this tool set.

### New mice expressing optogenetic actuators

We have characterized new transgenic mouse lines for photoinhibition via NpHR. Poor membrane trafficking of NpHR creates several problems, such as small photocurrent amplitude and ER distention (Gradinaru et al., 2010). Although neurons expressing this form of NpHR exhibit remarkable anatomical defects, they seem electrically normal and are capable of generating photocurrents (Zhao et al., 2008). Versions of NpHR with improved membrane trafficking (eNpHR2.0, eNpHR 3.0) avoid ER retention problems and yield significantly larger photocurrents (Gradinaru et al., 2010). Our transgenic mice expressing eNpHR2.0 exhibited the expected improvement in membrane targeting, as evidenced by the absence of ER swelling. However, we also made three surprising observations in these mice. First, maximum photocurrent amplitude (Figure 4B) was not increased over what has been reported for NpHR (Zhao et al., 2008) in the same type of neurons (cortical pyramidal cells) and with expression driven by the same promoter (Thy 1.2). Second, photocurrents mediated by eNpHR2.0 were activated at lower light intensities compared to photocurrents recorded in Thy1-NpHR mice (Figure 4B). Third, photocurrents mediated by eNpHR2.0 exhibited markedly faster activation and deactivation kinetics (Figure 4C). The reasons for these unexpected results are unclear but the latter two might arise as a consequence of improved intracellular trafficking of eNpHR2.0 in the new Thy1-eNpHR mice. The use of eNpHR3.0 might improve things even further and, in fact, a Cre-inducible eNpHR3.0 mouse has already been developed (Madisen et al., 2012). While photoinhibition is robust in this line, the Cre driver line strategy is limited because it can drive expression of only one optogenetic effector or reporter. Additionally, the requirement for mating two transgenic mouse lines can slow experiments. In such cases our eNpHR2.0 mice will be a valuable alternative.

With these new transgenic mice, we have shown that it is possible to reliably photoinhibit neuronal activity both *in vitro* (Figure 2) and *in vivo* (Figure 5) and to control motor behavior via light (Figure 5F) with sub-millimeter spatial resolution (Figures 5C,E). This performance enables many other photoinhibition applications. The red-shifted excitation spectrum of eNpHR2.0 is compatible with independent photostimulation via ChR2 (Gradinaru et al., 2008). It should be noted that we have excited ChR2 with 405 nm laser light, which falls short of the eNpHR excitation spectrum, while the 570 nm light we used to excite eNpHR2.0 is well beyond the excitation maximum for ChR2. Thus, the Thy1-eNpHR2.0 transgenic mice could be mated with ChR2 transgenic mice to permit bi-directional control of neuronal activity both *in vitro* and *vivo*.

We have also characterized several novel mouse lines useful for photostimulation. Thy1-VChR1 transgenic mice proved quite efficient for photostimulation of several types of neurons in various brain regions. In cortical pyramidal cells, VChR1-mediated photocurrents were quite substantial in amplitude (several hundred pA in P23 mice; Figure 7C), comparable to the largest photocurrents reported for the same type of neuron in Thy1-ChR2 mice (Wang et al., 2007). Furthermore, VChR1 appeared to be more light-sensitive than ChR2 even when using blue (465–495 nm) light for photostimulation (Figures 7E,F), potentially making the VChR1 mice more valuable than ChR2 mice for *in vivo* photostimulation applications. As reported previously, the kinetics of deactivation of VChR1 is substantially slower than the deactivation of ChR2, yielding a depolarization that persists for more than 100 ms after the end of a light flash (Figure 7I). This additional excitatory drive can yield extra action potentials that could be a limitation when using these mice for experiments where it is important to precisely control the timing, number, or frequency of action potentials. Nonetheless, the VChR1 mice were found to be quite useful for circuit analysis: with these mice, we could employ local photostimulation to map the spatial organization of synaptic connections in the NRTP (Figure 8F) and we are now using these mice for similar applications in other brain regions.

A similar type of trade-off can be seen in photostimulation with ChR2 vs. the ChR2 mutant H134R: while photocurrents mediated by ChR2-H134R deactivate more slowly than those produced by ChR2, ChR2-H134R photocurrents are somewhat larger in amplitude (Nagel et al., 2005; Gradinaru et al., 2007). We compared photostimulation properties in Purkinje cells expressing either ChR2 (PV) or ChR2-H134R (line 15) in BAC transgenic mice using the PV promoter to drive expression of these two ChR2 variants. In the cells expressing ChR2-H134R, photocurrent amplitude was somewhat larger, as expected (Figure 11E). However, the differences in deactivation kinetics are not a fatal flaw: because of the intrinsic electrical properties of Purkinje cells, even brief activation of ChR2 produces extra spikes (Figure 12A). Despite the potential for repetitive firing, optogenetic circuit mapping can be done both with the PV line (Figure 12F) as well as with line 15 (data not shown). Thus, for some applications kinetic properties are more important, while for others the magnitude of photostimulation is the main consideration. We have described mouse lines that are appropriate in either case.

### Fluorescent tagging of ChR2

It is useful to have optogenetic actuators with a wide range of fluorescent tags. For example, one channel of fluorescence emission can be used to visualize neuronal structure, while another can be used to define the spatial distribution of an optogenetic probe. Using tags of different colors also permits visualization of multiple types of actuators in different neuron types. For such reasons, we examined the effects of a total of three fluorescent tags (EYFP, mCherry, and tdTomato) on the function of ChR2 in transgenic mouse lines. Our results reveal that EYFP works well, while both mCherry and tdTomoto caused critical problems. EGFP tagging also seems to have no adverse effect on the function of Arch (Figures 11G,H and Madisen et al., 2012) or ChR2 (e.g., Katzel et al., 2011).

In Purkinje cells from PV-ChR2-mCherry mice, there was virtually no ChR2 present on the plasma membrane as evident in the absence of photocurrents in response to light. Instead, ChR2 appeared to be aggregated within the cytoplasm (Figure 9). This is remarkable, given that mCherry has been used successfully to tag ChR2 and other optogenetic probes in cultured neurons (Zhang et al., 2007), virus-injected mouse brains (Adamantidis et al., 2007; Zhang et al., 2007), and even *in vivo* in neurons differentiated from stem cells (Weick et al., 2010). However, aggregation has been reported in transgenic mice expressing mCherry fused to other proteins (Davidson and Campbell, 2009; Perry et al., 2009; Kremers et al., 2011) and there are suggestions of aggregation in transgenic mice expressing mCherry-tagged ChR2 and NpHR (see Figure 1E2 in Chuhma et al., 2011). Thus, aggregation of mCherry seems to occur, particularly when the mCherry fusion protein is chronically expressed at high levels.

Tagging ChR2 with tdTomato created a different set of issues: membrane trafficking of ChR2-tdTomato seemed adequate, because photocurrents could be detected in cortical pyramidal neurons expressing ChR2-tdTomato. However, neurons expressing the highest levels of ChR2-tdTomato seemed fragile, as evident by leakiness after establishing whole-cell recording conditions. This was a consistent finding observed in laboratories in 2 different countries and might help account for previous observations that neurons expressing ChR2-tdTomato are more heterogeneous in their sensitivity to light and tend to be less sensitive to photostimulation (Madisen et al., 2012). The reason for the apparent fragility of neurons expressing ChR2-tdTomato is not clear, but might be related to the tendency of tdTomato to dimerize (Shaner et al., 2004, 2007). Whatever the reasons for these problems, for purposes of expression in transgenic mice it seems advisable to avoid tagging ChR2 (and other optogenetic probes) with mCherry or tdTomato. Alternatively, it may be possible to use bi-cistronic strategies, such as IRES or 2a sequences (Tang et al., 2009; Katzel et al., 2011), to express both ChR2 and fluorophores as separate proteins.

### Molecular genetic expression strategies

Recent advances in transgenic mouse technology and optogenetic probe development provide various approaches for cell-specific control of neurons in the mouse brain (Hägglund et al., 2010; Zhao et al., 2011; Madisen et al., 2012; Tanaka et al., 2012). We performed a side-by-side comparison of three transgenic mouse lines that express ChR2 in cerebellar Purkinje cells. Our analysis demonstrated clear differences between these lines, both in the degree of cell-type specificity of ChR2 expression and in photostimulation capabilities. Our conclusion was that targeting based on Purkinje cell-type expression of Cre recombinase plus the use of a mouse line expressing a Cre-inducible ChR2-H134R yield expression exclusively in Purkinje cells and largest photocurrents at a given light intensity (Figure 11). It is possible that future mouse lines incorporating Cre-indicible versions of even newer ChR2 variants could improve functionality still further (Gunaydin et al., 2010; Berndt et al., 2011; Kleinlogel et al., 2011). Furthermore, our results confirm the versatility of the Cre/lox strategy for expression of other optogenetic probes, such as Arch, in Purkinje cells and suggest a broad utility for targeting ChR2 expression to other genetically-defined neuronal populations. Indeed, the availability of many hundreds of Cre driver lines should greatly facilitate the generation of transgenic mice expressing optogenetic probes in almost any neuron of interest.

### Functional connectomics

The mouse brain consists of approximately 10^11^ neurons of hundreds of different types that form ~10^14^ synaptic connections (Marois and Ivanoff, 2005). In recent years it has been proposed that this complete collection of synaptic connections—the connectome—might be fully described (Lichtman et al., 2008; see also Sporns et al., 2005 for the human brain). While most current efforts to characterize the mouse brain connectome are based on high-content anatomical analyses (Smith, 2007; Lichtman and Denk, 2011), it is also possible to envision a functional analysis based on ChR2-mediated photostimulation (Luo et al., 2008; Augustine et al., 2012). For this purpose, we have summarized the patterns of expression of such probes for some of the lines that are characterized in the present paper, as well as in previous work done as part of the collaboration between the Augustine, Deisseroth and Feng labs (Table 2). This analysis is based on histological observation of fluorescently-tagged probes in the cell bodies of the indicated neurons, as well as confirmation of functionality in some cases. This summary makes clear that on the order of 100 different types of neurons are now addressable via optogenetic photostimulation, meaning that the spatial organization of their circuits can be characterized using the local photostimulation approach depicted in Figures 8, 13. This already represents a significant fraction of all neuron types found in the mouse brain and it is reasonable to imagine that the remaining cell types could be covered by mating existing Cre driver lines to floxed ChR2 lines, as exemplified in Figures 11–13. Recent improvements in optogenetic approaches for imaging neuronal activity (Kralj et al., 2011; Baker et al., 2012; Jin et al., 2012; Knopfel, 2012; Akerboom et al., 2013; Grimley et al., 2013; Marvin et al., 2013) promise to further accelerate mapping throughput (Luo et al., 2008; Mancuso et al., 2011; Augustine et al., 2012).

In conclusion, we have characterized several new lines of transgenic mice that will aid optogenetic analysis of brain function at the level of individual neurons, synaptic circuits, and the entire connectome. Most of these mice are commercially available (Table 1) and express a variety of optogenetic probes in many different types of neurons (Table 2). In addition, our efforts indicate some pitfalls associated with transgenic expression of fluorescently tagged optogenetic probes and compare several different molecular genetic strategies for transgenic expression of optogenetic probes in mouse neurons.

### Conflict of interest statement

The authors declare that the research was conducted in the absence of any commercial or financial relationships that could be construed as a potential conflict of interest.
